# Comparative Transcriptome Analysis of Key Genes and Pathways Activated in Response to Fat Deposition in Two Sheep Breeds With Distinct Tail Phenotype

**DOI:** 10.3389/fgene.2021.639030

**Published:** 2021-04-08

**Authors:** Wei Zhang, Mengsi Xu, Juanjuan Wang, Shiyin Wang, Xinhua Wang, Jingquan Yang, Lei Gao, Shangquan Gan

**Affiliations:** ^1^State Key Laboratory of Sheep Genetic Improvement and Healthy Production, Xinjiang Academy of Agricultural and Reclamation Sciences, Shihezi, China; ^2^Xinjiang Agricultural Vocational Technical College, Changji, China

**Keywords:** Altay sheep, tail fat deposition, transcriptome, gene expression, RNA-Seq

## Abstract

Fat tail in sheep presents a valuable energy reserve that has historically facilitated adaptation to harsh environments. However, in modern intensive and semi-intensive sheep industry systems, breeds with leaner tails are more desirable. In the present study, RNA sequencing (RNA-Seq) was applied to determine the transcriptome profiles of tail fat tissues in two Chinese sheep breeds, fat-rumped Altay sheep and thin-tailed Xinjiang fine wool (XFW) sheep, with extreme fat tail phenotype difference. Then the differentially expressed genes (DEGs) and their sequence variations were further analyzed. In total, 21,527 genes were detected, among which 3,965 displayed significant expression variations in tail fat tissues of the two sheep breeds (*P* < 0.05), including 707 upregulated and 3,258 downregulated genes. Gene Ontology (GO) analysis disclosed that 198 DEGs were related to fat metabolism. In Kyoto Encyclopedia of Genes and Genomes (KEGG) pathway analysis, the majority of DEGs were significantly enriched in “adipocytokine signaling,” “PPAR signaling,” and “metabolic pathways” (*P* < 0.05); moreover, some genes were involved in multiple pathways. Among the 198 DEGs, 22 genes were markedly up- or downregulated in tail fat tissue of Altay sheep, indicating that these genes might be closely related to the fat tail trait of this breed. A total of 41,724 and 42,193 SNPs were detected in the transcriptomic data of tail fat tissues obtained from Altay and XFW sheep, respectively. The distribution of seven SNPs in the coding regions of the 22 candidate genes was further investigated in populations of three sheep breeds with distinct tail phenotypes. In particular, the g.18167532T/C (Oar_v3.1) mutation of the ATP-binding cassette transporter A1 (*ABCA1*) gene and g.57036072G/T (Oar_v3.1) mutation of the solute carrier family 27 member 2 (*SLC27A2*) gene showed significantly different distributions and were closely associated with tail phenotype (*P* < 0.05). The present study provides transcriptomic evidence explaining the differences in fat- and thin-tailed sheep breeds and reveals numerous DEGs and SNPs associated with tail phenotype. Our data provide a valuable theoretical basis for selection of lean-tailed sheep breeds.

## Introduction

Fat tail is a valuable trait that helps sheep (*Ovis aries*) adapt to harsh conditions, such as extremely cold winters, food shortages, and drought seasons. Fat-tailed sheep characteristically deposit a mass of fatty tissue in the tail region during summer and autumn seasons when nutritious pastures are available. In winter when temperatures are extremely low and grasslands are covered with heavy snow for long periods of time, these animals obtain energy by decomposing the fat deposits in their tails to sustain life (Moradi et al., 2012; Gan et al., 2013a). According to archaeological and genomic findings, modern sheep breeds were domesticated at the Fertile Crescent region of Iraq about 9,000 years ago (Zeder, 1999; Handley et al., 2007; Chessa et al., 2009). Similar to their Asian mouflon ancestor, modern sheep breeds were thin tailed at the early stages. Fat-tailed sheep appeared ∼5,000 years ago through long-term artificial and natural selection during the long evolution process to adapt to harsh local climate conditions (Cooper, 1986; Wade et al., 2010; Muigai and Hanotte, 2013; Beynon et al., 2015). However, in modern society, dietary habits and health concepts have undergone a profound revolution, and mutton with lower fat content is preferred for consumption. Furthermore, the fat deposition requires more energy than the growth of lean tissues. Thus, the efficiency of meat production is higher than that of fat in intensive and semi-intensive sheep industry systems, and the tail fat, which constitute about 20% of the total carcass weight, markedly reduce their economic value (Nejati-Javaremi et al., 2007). For the above reasons, the fat-tailed sheep breeds are gradually becoming less preferable by producers and consumers, and lean-tailed sheep breeds are more desirable (Momani-Shaker et al., 2002; Gokdal et al., 2004; Kashan et al., 2005; Unal et al., 2006; Khaldari et al., 2007; Marai et al., 2009). Therefore, elucidation of the key genes that regulate the deposition and decomposition of tail fat in sheep and the molecular mechanisms controlling fat metabolism would greatly accelerate the breeding course of lean-tailed sheep and production of more healthy mutton to the market.

Fat is not only used for energy storage but is also an important endocrine tissue involved in regulating crucial physiological and biochemical reactions in organisms (Scherer, 2006; Galic et al., 2010; Poulos et al., 2010; Ohashi et al., 2011). Traditionally, research on the molecular regulatory mechanisms of sheep tail adipose tissue development and deposition has only involved the investigation of the functions of single candidate genes. Earlier, Kumar et al. (1998) reported higher expression of the leptin (*LEP*) gene in perirenal, backside, and omental fat tissues of fat genotype than lean genotype Coopworth sheep. Both activity and expression of lipoprotein lipase (*LPL*) gene were increased in fat tissue of the fat genotype sheep group. Moreover, its expression was tissue specific but not affected by the nutrition level (Bonnet et al., 2000). In Guangling large-tailed sheep, the uncoupling protein 1 (*UCP1*) gene was expressed at significantly higher levels in perirenal fat than other tissues but showed very low expression in subcutaneous fat (Yuan et al., 2012). The cell death-inducing DFFA-like effector c (*CIDEC*) gene was highly expressed in rump fat tissue of Altay sheep, which decreased significantly after a 4-week fasting period (Gao et al., 2015). Expression of fatty acid binding protein 4 (*FABP4*) gene was significantly elevated in tail fat of Lori-Bakhtiari, a fat-tailed sheep breed, compared with Zel, a thin-tailed sheep breed (Bakhtiarizadeh et al., 2013). In Altay sheep, *FABP4* gene was abundantly expressed in intestinal and rump fat tissues and showed no significant changes when the nutritional status of sheep altered, suggesting its fundamental role in adipose metabolism (Xu et al., 2015). The collective findings highlight the involvement of several critical genes in the regulation of tail fat deposition and mobilization in sheep. In general, traits of animals, including tail size, are modulated by multiple interconnected genes that form a refined regulatory network to manage complex internal and external environments. Therefore, research specifically focusing on one or several genes cannot completely elucidate such a network.

In recent years, due to the rapid development of next-generation sequencing and application of RNA sequencing (RNA-Seq) technology, numerous genes expressed in tail fat tissue of sheep have been identified *via* analysis of transcriptome data in attempts to clarify the molecular network regulating adipose deposition and metabolism in fat tail of sheep. RNA-Seq has been used successfully to investigate the genes expressed in fat tissue of sheep. Wang et al. (2014) applied RNA-Seq technology to compare the transcriptome profiles of two sheep breeds, Kazak (fat-tailed) and Tibetan (short-tailed). Their study led to the identification of 646 differentially expressed genes (DEGs) between the two sheep breeds, including 280 upregulated and 366 downregulated genes. Moreover, the genes NEL-like 1 (*NELL1*) and flavin containing monooxygenase 3 (*FMO3*), which displayed the most significant fold changes, were highly correlated with adipose deposition in the tail (Wang et al., 2014). Guangling large-tailed sheep and small-tailed Han sheep are two typical fat-tailed sheep breeds in China. Using RNA-Seq, a total of 4,131 DEGs were determined in tail fat tissues of these two breeds, with *FABP4*, fatty acid binding protein 5 (*FABP5*), adiponectin (*ADIPOQ*), and cluster of differentiation 36 (*CD36*) identified as the four most highly transcribed genes (Li et al., 2018). A research on small-tailed Han and Dorset sheep revealed 602 DEGs, and Gene Ontology (GO) analysis showed that several of these genes were enriched in the triglyceride biosynthetic process (Miao et al., 2015). By applying RNA-Seq, Ma et al. (2018) investigated the tail fat transcriptome of Lanzhou fat-tailed (long fat-tailed), small-tailed Han (short fat-tailed), and Tibetan (short thin-tailed) sheep and identified several DEGs and long non-coding RNAs (lncRNAs). GO and pathway analysis of DEGs and target genes of differentially expressed lncRNAs revealed that the majority were enriched in fatty acid metabolism and fatty acid elongation-related pathways that contribute to fat deposition (Ma et al., 2018). Previous studies clearly indicate that the tail fat deposition ability of sheep with different tail phenotypes is a complex quantitative trait regulated by multiple genes. The molecular mechanisms underlying tail fat deposition remain to be elucidated.

**FIGURE 1:**
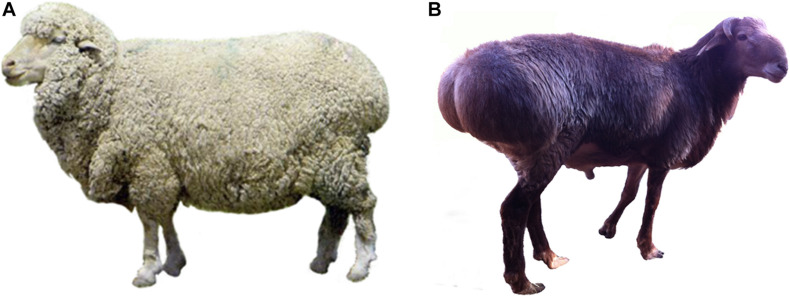
Two sheep breeds used in the current investigation. **(A)** Thin-tailed Xinjiang fine wool (XFW) sheep; **(B)** fat-rumped Altay sheep. The distinct tail phenotypes of the two sheep breeds used in the current study are shown here (photos are taken by authors).

Due to the multiple functional roles of fat tissues (Scherer, 2006; Galic et al., 2010; Poulos et al., 2010; Ohashi et al., 2011), the fat deposition in the animal’s body is most likely to be affected by various factors, such as environment temperature, food, and breeds. Thus, to systematically investigate the relationship between the fat deposition ability and genetic difference of sheep, such influencing factors should be taken into consideration. Altay and Xinjiang fine wool (XFW) sheep are both distributed in the Xinjiang province of China for a long time in history, and their tail fat deposition ability is extremely distinct. Altay sheep is one of the most popular fat-tailed breeds. Their tail and rump are fused together, and they are characterized by their ability to deposit rump fat. Our research showed that the rump fat weight of adult male Altay sheep accounted for about ∼25% of carcass weight on average during autumn (the data have not yet been published). In contrast, XFW sheep, a typical long thin-tailed sheep breed, stores almost no fat tissue in its tail. Thus, these two sheep breeds with distinct tail fat characteristics represent good models for investigating DEGs involved in the regulation of tail fat deposition (Figure 1). Accordingly, we focused on rump and tail fat tissues of Altay and XFW sheep as model animals. RNA-Seq technology was applied to identify DEGs and the associated signaling pathways, with the aim of highlighting candidate genes and mechanisms that play critical roles in regulating adipose deposition in tail of sheep. Genetic variations of these DEGs were also further investigated. Our collective data would provide fundamental information and theoretical guidelines for efficient breeding of lean-tailed sheep.

## Materials and Methods

### Ethics Approval and Consent to Participate

All animal experimental procedures were approved by the Biological Studies Animal Care and Use Committee, Xinjiang Production and Construction Corps, Peoples Republic of China, and the ethics committee of Xinjiang Academy of Agricultural and Reclamation Sciences, People’s Republic of China approved this study (Permit Number: XAARS-AE-2018012).

### Tail Fat Collection and RNA Extraction

Three healthy male Altay and XFW sheep (3–4 years of age) were randomly selected, respectively, from the sheep farm in the Animal Husbandry Institute of Xinjiang Academy of Agricultural and Reclamation Sciences, Shihezi, Xinjiang, China. The body weight of Altay and XFW sheep were 105 ± 5 and 100 ± 5 kg, respectively, and the individuals of each sheep breed were randomly selected to ensure that they did not have a genetic relationship. The sheep were reared according to the feeding standard of meat-producing sheep and goats of China (NY/T 816-2004). At the morning, noon, and afternoon of each day, sheep were provided sufficient forage and clear water. Each evening, an additional 250 g corn per animal was supplied. Six months later, the rump region of Altay sheep were full and round owing to massive fat tissue deposition while no obvious fat tissue were present in tail and rump of XFW sheep. Before sampling, the sheep were anesthetized by injecting 30 mg/kg body weight of pentobarbital sodium (Ningbo, Zhejiang, China) through ear vein (Yan et al., 2013). The pentobarbital sodium is a kind of medium-efficiency barbital hypnotics that could inhibit the uplink activation system of the brain stem reticular structure. Five minutes later, the sheep were slaughtered when they were under anesthesia status, and 100 g of tail fat tissue from each sheep were rapidly collected. The fat tissues were collected from the left rump of Altay sheep, and from the whole tail of XFW sheep due to their very little tail fat. Samples were immersed in liquid nitrogen for transportation and maintained at –80°C in the laboratory.

Total RNA was extracted from tail adipose tissue using TRIzol reagent (Invitrogen, California, United States) according to the manufacturer’s protocol. In short, the fat tissue was homogenized using homogenizer in TRIzol reagent and centrifuged for 5 min at 12,000 × *g* at 4°C. The clear supernatant was transferred to a new tube and incubated for 5 min to allow complete dissociation of the nucleoproteins complex. Then the chloroform was added, and the mixture was mixed thoroughly by shaking the tube. Subsequently, the tube was incubated for 2–3 min and centrifuged for 15 min at 12,000 × *g* at 4°C. The aqueous phase containing the total RNA was transferred to a new tube, then isopropanol was added and the mixture was centrifuged for 10 min at 12,000 × *g* at 4°C; the total RNA precipitate forms a white gel-like pellet at the bottom of the tube. The concentration and integrity of total RNA were evaluated using the 2100 Bioanalyzer instrument (Agilent Technologies, Waldronn, Germany) by measuring absorbance at 260 and 280 nm.

### Blood Sample Collection and Genomic DNA Extraction

The fat-rumped Altay sheep, thin-tailed XFW sheep, and short fat-tailed Hu sheep were selected to investigate the distribution of single nucleotide polymorphisms (SNPs) in different sheep populations with distinct tail phenotypes. A total of 104 individuals of each sheep breed were randomly selected, and 5 ml venous anticoagulation blood was collected from each sheep through the anterior vena cava. Genomic DNA was extracted from blood samples using the Blood DNA Extraction kit (Tiangen, Beijin, China). DNA concentration and purity were evaluated using the 2100 Bioanalyzer instrument (Agilent Technologies, Waldronn, Germany). The OD_260_/OD_280_ value of DNA samples was ∼1.8. DNA was dissolved in TE buffer (pH = 8.0), and the concentration was adjusted to 200 ng/μl, then stored at –20°C before being used to detect SNPs in the sequences of candidate genes.

### The cDNA Library Construction and Sequencing

Total RNA from three Altay sheep was mixed to generate a cDNA library and similarly from three XFW sheep to generate another cDNA library. After treatment of total RNA with DNase I, poly(A) mRNA was isolated using oligo (dT) magnetic beads (Invitrogen, California, United States). Then the isolated mRNA was cut into short fragments in fragmentation buffer, and the first-strand cDNA was synthesized using random hexamer primers and reverse transcriptase. Subsequently, second-strand cDNA was synthesized, purified using the QiaQuick PCR extraction Kit (QIAGEN, Hilden, Germany), and the poly(A) fragment was added to both ends. The short fragments were connected with sequencing adapters and separated on gels *via* electrophoresis. Suitable fragments were selected as templates for amplification to construct cDNA libraries. Finally, the two libraries were paired-end sequenced using Illumina HiSeq 2000 at the Beijing Genomics Institute (Shenzhen, China).

### Analysis of Sequencing Data

Applying trimmomatic (Bolger et al., 2014), the adapter sequences, reads in which the percentage of low-quality bases (quality value *Q* ≤ 10) were > 50%, and reads in which unknown bases were >2% were filtered to avoid disturbing the subsequent assembling and analysis (Li et al., 2018). Clean reads were imported into FastQC using FastQ format for further quality control and sequencing quality evaluated (Brown et al., 2017). After that, the clean reads were aligned against the sheep reference genome (*Ovis aries*, v3.1) using Spliced Transcripts Alignments to a Reference (STAR) (Dobin et al., 2013), and then assembled using StringTie (Kovaka et al., 2019) by comparing with the reference genome of *Bos taurus* (v3.1) and *Ovis aries* (v3.1). Meanwhile, GffCompare was used to test the sensitivity and precision of comparison result (Pertea and Pertea, 2020). Finally, the assembled reads were annotated based on protein databases. To get comprehensive annotation information, NR (RefSeq non-redundant proteins) (Pruitt et al., 2007), Swiss-Prot (Boutet et al., 2016), and Cluster of Orthologous Groups of proteins (COG) (Tatusov et al., 2001) databases were used simultaneously.

Expression levels of genes were determined based on gene coverage and fragments per kilobase million (FPKM) values. The gene coverage value represents the ratio of the number of bases on the unigene covered by reads and unigene sequence length and FPKM represents fragments per kilobase of transcript per million reads mapped (Mortazavi et al., 2008; Li et al., 2018). The FPKM method could be used to eliminate the influence of different gene lengths and sequencing levels on calculation of gene expression, allowing direct comparison of gene expression differences between two samples. Expression levels of genes were subsequently normalized and the ratios calculated using Cufflinks^1^. The cuffdiff module of cufflinks was applied to identify DEGs between two samples (Trapnell et al., 2013). The *t*-test was used to get the *P*-value of unigenes. The relative expression of unigenes in tail fat tissues of Altay and XFW sheep was determined according to log_2_fat-rump-ratio/log_2_thin-tail-ratio, and DEGs screened taking log_2_ratio ≥ 1 or ≤ –1 and *P* < 0.05 as standard criteria. Finally, multiple hypothesis testing was applied to revise the *P*-value of each DEG to guarantee the low false discovery rate in whole. We filtered out DEGs for subsequent analyses by setting the false discovery rate (FDR) as ≤ 0.001 and absolute value of log_2_ratio ≥ 2 as the threshold.

SNPs of different transcriptomes were further detected using the Genome Analysis Toolkit (GATK) software package (v4.0.10) (Franke and Crowgey, 2020). Based on comparison of short sequences, SNP sites were filtered using the Short Oligonucleotide Alignment Program (SOAPsnp) package to obtain high-quality variants and the conditions set as variation detection quality ≥ 30, site depth 10–100 × , and adjacent SNPs > 10 bp (Li et al., 2008).

### GO and Kyoto Encyclopedia of Genes and Genomes Analyses of DEGs

GO analysis was employed to annotate functions and further enriching the GO terms of DEGs using David online software and DEGs were mapped to GO terms in the database^2^. Gene numbers for each term were ultimately calculated. Significantly enriched GO terms for DEGs were determined using a hypergeometric test (Cao and Zhang, 2014). Next, the *P*-value was subjected to Bonferroni correction and threshold *P* < 0.05 used to define significantly enriched GO terms for DEGs. Meanwhile, the Kyoto Encyclopedia of Genes and Genomes (KEGG) database^3^ was applied for pathway enrichment analysis of DEGs. Pathways with *P* < 0.05 were considered significantly enriched for DEGs.

### Quantitative Real-Time PCR Validation of RNA-Seq Results

Based on the results from GO, KEGG, and SNP detection of DEGs, 22 candidate genes related to tail fat deposition were selected, including 11 upregulated and 11 downregulated genes. Using non-pooled RNA samples (*n* = 3 for each breed), the expression patterns of these genes in tail fat tissues of Altay and XFW sheep were examined *via* quantitative real-time PCR (qRT-PCR) to validate the reliability of RNA-Seq data. Beta-actin (*β-actin*) gene served as the internal control gene. qRT-PCR primers were designed using Oligo6.0 and synthesized by Sangon Biotech Co., Ltd. (Shanghai, China). The 22 genes and their primers are listed in Supplementary Table 1.

Using the PrimeScript II 1st-Strand cDNA Synthesis Kit (Takara, Dalian, China), cDNAs for detection of DEGs involved in tail fat deposition were generated *via* qRT-PCR conducted on a Roche 480 instrument (Roche, Mannheim, Germany) using SYBR Green PCR Master Mix kit (QIAGEN, Germany) according to the manufacturer’s instructions. The reaction mixture comprised 10 μl 2 × Quanti Fast SYBR Green PCR Master Mix, 1 μl cDNA (<100 ng), 0.5 μl forward and reverse primers (10 μM), respectively, and ddH_2_O to a total volume of 20 μl. The following conditions were used for amplification: 95°C for 5 min, followed by 45 cycles of 95°C for 10 s, 65°C for 30 s, and 72°C for 7 min. qRT-PCR analysis was performed in triplicate for each sample and relative expression for each gene estimated with the 2^–△^^△^^*Ct*^ method. Data were analyzed using the Statistical Analysis System (version 6.12; SAS Institute, Inc., Cary, NC, United States) and results expressed as means ± SD. Significance of differences was analyzed using one-way analysis of variance (ANOVA). Differences were considered significant at *P* < 0.05 and highly significant at *P* < 0.01.

### Analysis of Variations in Candidate Functional Genes Involved in Fat Deposition

Based on SNP analysis of candidate genes, 10 SNPs existing in coding regions of the 22 candidate functional genes involved in tail fat deposition were selected for analysis. Sequences containing the selected SNP sites were exported from transcriptome data and mapped onto the sheep genome (ISGC Oar_v3.1/oviAri3, August 2012) by applying Blat of the UCSC Genome Browser^4^, and subsequently 1,000 bp sequences around the SNP sites were cut out to design primers using Oligo 6.0 (Supplementary Table 2). The PCR fragment sizes were 200–300 bp, and SNP sites were located near the middle of the amplified fragments. Primers were synthesized by Sangon Biotech Co., Ltd. (Shanghai, China).

Genomic DNA of Altay (*n* = 104), Hu (*n* = 104), and XFW (*n* = 104) sheep were amplified using the above primers (Supplementary Table 2), and the distribution of the 10 SNPs in these three breeds were determined *via* single-strand conformation polymorphism analysis of PCR-amplified fragments (PCR-SSCP) or restriction fragment length polymorphism analysis of PCR-amplified fragments (PCR-RFLP) technology. The PCR reaction mixture comprised 2.5 μl 10 × PCR buffer, 0.5 μl DNA template (100 ng), 2 μl dNTPs (2.5 mM each), 0.5 μl forward and reverse primers (10 μM), and 0.5 μl *Taq* DNA polymerase (5 U/μl) (TaKaRa, Dalian, China), with ddH_2_O to a total volume of 25 μl. The following conditions were used for amplification: 95°C for 5 min; 45 cycles of 95°C for 30 s, 55°C for 30 s, and 72°C for 30 s; and 72°C for 10 min. Amplified products were detected *via* 1.5% agarose gel electrophoresis and used for SSCP or RFLP analysis. Then the distributions of SNPs in populations of three sheep breeds were calculated, and the relationship between the SNP and tail fat deposition ability was determined by its genotype frequency. If the genotype frequency of a SNP was significantly different in three sheep breeds (*P* < 0.05), we speculated it might relate to tail fat deposition ability. The Hardy-Weinberg equilibrium of SNPs in the three groups was verified by calculating the expected frequencies and numbers, and tested using the goodness-of-fit χ^2^ test.

### Interaction Analysis of Candidate Proteins Involved in Adipose Metabolism

Search Tool for the Retrieval of Interacting Genes (STRING/Proteins) 11.0 (Szklarczyk et al., 2019) was applied to analyze the protein-protein interactions of candidate genes involved in adipose metabolism with the threshold score > 0.4 (medium confidence), and then the interaction network was illustrated.

## Results

### Summary of Transcriptome Sequencing Data

Two cDNA libraries were constructed using mRNAs extracted from fat-rumped Altay sheep and thin-tailed XFW sheep, sequenced, and two sets of raw reads were obtained containing 51,943,518 and 51,770,440 raw reads, respectively. Low-quality raw reads and adapter sequences were then filtered, ultimately resulting in 46,614,192 and 46,646,110 clean reads. Approximately 84 and 81% clean reads could be mapped to the sheep reference genome (*Ovis aries*, v3.1). The clean reads were finally assembled into Unigenes, which were categorized to two classes, specifically, clusters and singletons. Clusters were labeled by the prefix “CL,” followed by the cluster id. A single cluster included several Unigenes with > 70% sequence similarity. Singletons were indicated by the prefix “Unigene” (Supplementary Table 3). In total, 153,914 and 117,254 clusters and 78,065 and 56,293 singletons were obtained from the two sample sets, respectively. The mean lengths of clusters were 335 and 317 nt, while mean lengths of singletons were 696 and 629 nt for Altay and XFW sheep groups, respectively (Supplementary Figure 1). Clusters and singletons were further analyzed and filtered, resulting in a final total of 48,894 Unigenes. Transcriptome sequencing data are summarized in Table 1.

**TABLE 1:** Transcriptome sequencing data from Altay and Xinjiang fine wool (XFW) sheep.

Samples	Altay	XFW
Total raw reads	51,943,518	51,770,440
Total clean reads	46,614,192	46,646,110
Total clean nucleotides (nt)	4,661,419,200	4,664,611,000
Q20 percentage (%)	97.97	97.86
N percentage (%)	0.01	0.01
GC percentage (%)	48.41	47.15
Error rate (%)	0.01	0.01
Total mapped reads	39,155,921	37,783,349
Multiple mapped reads	1,370,457	1,435,767
Unique mapped reads	37,785,464	36,347,582
Unmapped reads	7,458,280	8,862,761
Mapping rate (%)	84	81
Total number of clusters	153,914	117,254
Total number of singletons	78,065	56,293
Total length of clusters (nt)	51,601,654	37,173,312
Total length of singletons (nt)	54,302,714	35,416,372
Mean length of clusters (nt)	335	317
Mean length of singletons (nt)	696	629
N50 length of clusters (nt)	573	519
N50 length of singletons (nt)	1,114	917

### Annotation and Expression Analysis of Unigenes

Comparison of the Unigenes obtained with known gene sequences of *Bos taurus* (v3.1) and *Ovis aries* (v3.1) revealed a total of 21,527 genes (*E*-value < 0.00001), which were subsequently matched to NR, Swiss-Prot, KEGG, and COG, leading to 57, 53, 61, and 45% annotation, respectively (*E*-value < 0.00001). GO analysis was applied to clarify the biological functions of the above genes (Supplementary Table 3).

**FIGURE 2:**
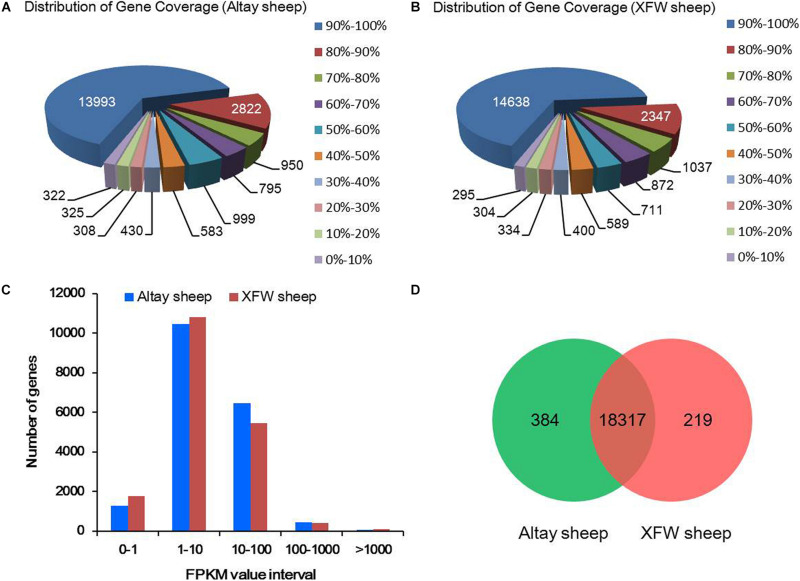
The summary of RNA-Seq data analysis. **(A,B)** Distribution of gene coverage in Altay and Xinjiang fine wool (XFW) sheep groups. **(C)** Numbers of annotated genes with different expression levels against a range of fragments per kilobase million (FPKM) values. **(D)** Venn diagram of unique and shared genes in the tail fat tissues of Altay and XFW sheep.

Calculation of gene coverage revealed that 65% (13,993/21,527) genes of Altay sheep and 68% (14,638/21,527) genes of XFW sheep had 90–100% coverage (Figures 2A,B). In total, 19,878 annotated genes with FPKM > 0 were detected in the two samples. The FPKM trends of the two samples were comparable, indicating similar expression patterns of the majority of genes in tail fat tissues of Altay and XFW sheep (Figure 2C). The largest proportions of genes were expressed at low (1 < FPKM < 10) and moderate (10 < FPKM < 100) levels and only a small fraction expressed at high levels (FPKM > 100). The results indicate that high-throughput sequencing technology has an obvious advantage in detection of low-abundance genes. Further analysis revealed 94.08% (18,701/19,878) of the total genes, including 384 uniquely expressed genes, in the fat rump of Altay sheep and 93.25% (18,536/19,878) of total genes, including 219 uniquely expressed genes, in the thin tail of XFW sheep. Overall, we detected 18,317 common genes in the tail fat tissues of the Altay and XFW sheep (Figure 2D).

### Analysis of DEGs Between Tail Fat Tissues of the Two Sheep Breeds

**FIGURE 3:**
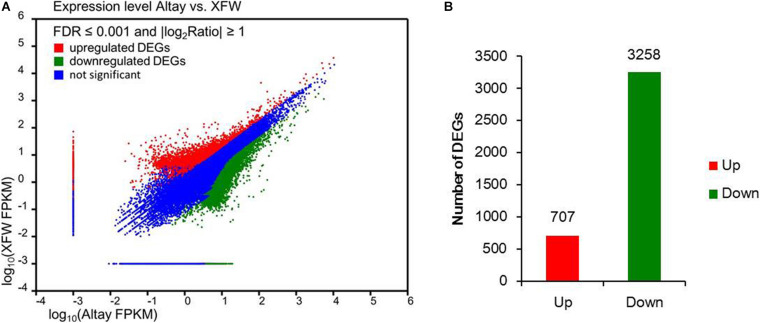
The differentially expressed genes (DEGs) in tail fat of two sheep breeds. **(A)** Expression levels of genes detected in tail fat tissues from Altay and Xinjiang fine wool (XFW) sheep. **(B)** Up- and downregulated genes in tail fat tissue of Altay compared with XFW sheep.

In total, 8,042 DEGs were identified between the two sheep breeds using FDR ≤ 0.001 and | log_2_ratio| ≥ 1 as filter criteria (Figure 3A and Supplementary Table 4). Within this gene set, differences in levels of 3,965 DEGs in tail fat tissues of the two sheep breeds were highly significant (FDR ≤ 0.001 and | log_2_ratio| ≥ 2), including 707 highly upregulated and 3,258 highly downregulated genes in fat-rumped Altay sheep, compared to thin-tailed XFW sheep (Figure 3B and Supplementary Table 4).

To further clarify the functions of DEGs in tail fat metabolism of the two sheep breeds, we identified 198 DEGs (72 upregulated and 126 downregulated) closely related to adipose tissue development, deposition, and mobilization. Among these genes, the expression levels of *ABCA1*, *PLIN1*, *SORBS1*, *ANGPTL4*, *LPIN1*, *ELOVL5*, *ACACA*, *FASN*, *CIDEC*, *FABP3*, and *SLC27A2* were significantly higher in Altay than XFW sheep. In contrast, *CYP4A11*, *FADS2*, *PTPLB*, *ACAA1*, *PPCK1*, *PMP2*, *HSL*, *CPT1A*, *C1QTNF1*, *ACADL*, and *C1QTNF9* were more highly expressed in tail fat of XFW than Altay sheep (Supplementary Table 4).

Based on significant differences in expression of these genes between tail fat tissues of Altay and XFW sheep breeds and their participation in regulation of fat metabolism, we speculate that the DEGs identified play potentially important roles in influencing tail phenotypes of different sheep breeds. We further focused on 22 DEGs showing highly significant up- or downregulation in tail fat tissue of Altay sheep as candidate genes.

### qRT-PCR Validation of RNA-Seq Data

**FIGURE 4:**
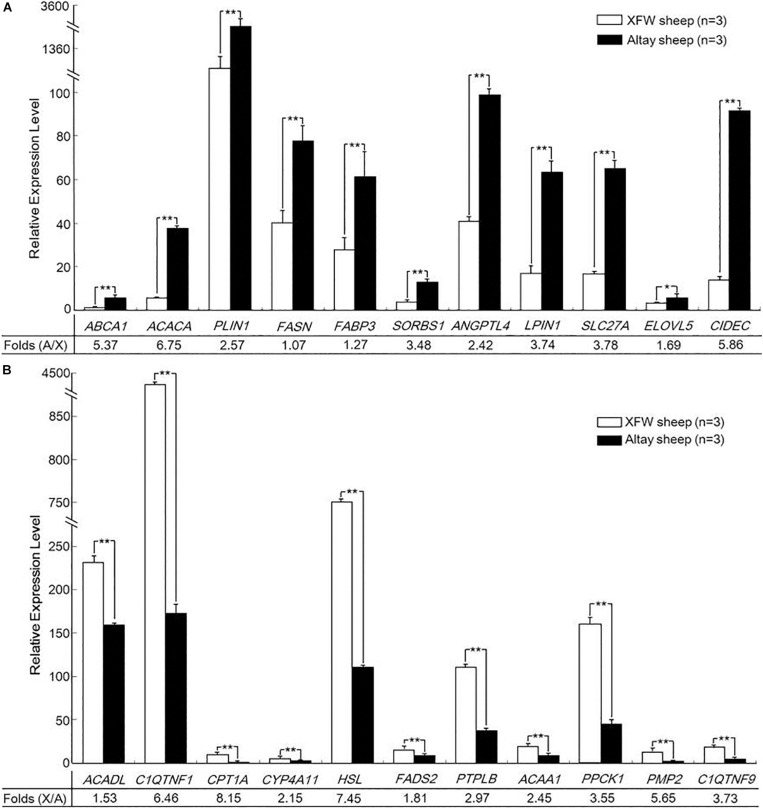
Validation of the differentially expressed genes (DEGs) by qRT-PCR. **(A)** The expression levels of 11 upregulated genes. **(B)** The expression levels of 11 downregulated genes in tail fat tissue of Altay relative to Xinjiang fine wool (XFW) sheep (*P < 0.05, **P < 0.01).

The qRT-PCR expression patterns of the investigated genes were consistent with RNA-Seq data (Figure 4 and Supplementary Figure 2), supporting the reliability of the expression profile generated with RNA-Seq.

Among the 22 candidate genes, 10 genes (*ABCA1*, *ACACA*, *PLIN1*, *FASN*, *FABP3*, *SORBS1*, *ANGPTL4*, *LPIN1*, *SLC27A*, and *CIDEC*) were highly upregulated in rump fat tissue of Altay sheep relative to tail fat tissue of XFW sheep (*P* < 0.01). In particular, the expression of *ACACA*, *ABCA1*, and *CIDEC* in rump fat of Altay sheep was 6.75, 5.37, and 5.86 times higher than that in tail fat of XFW sheep (Figure 4A). Eleven other genes (*ACADL*, *C1QTNF1*, *CPT1A*, *CYP4A11*, *HSL*, *FADS2*, *PTPLB*, *ACAA1*, *PPCK1*, *PMP2*, and *C1QTNF9*) were more highly expressed in tail fat tissue of XFW than Altay sheep (*P* < 0.01), particularly *HSL* and *CPT1A*, which were 7.45 and 8.15 times higher, respectively (Figure 4B). In view of these findings, we hypothesized that these genes might play potential roles in regulating tail fat metabolism of Altay and XFW sheep and ultimately influence their tail phenotypes.

### GO and KEGG Analyses of DEGs Between Altay and XFW Sheep

**FIGURE 5:**
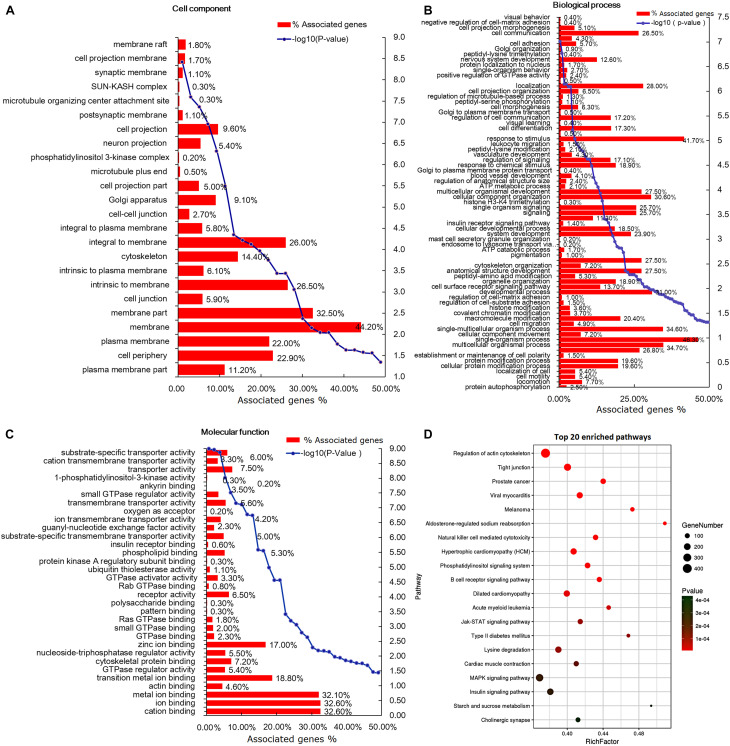
Gene ontology (GO) and Kyoto Encyclopedia of Genes and Genomes (KEGG) analysis of the differentially expressed genes (DEGs). **(A)** Cell component-based classification of DEGs. **(B)** Biological process-based classification of DEGs. **(C)** Molecular function-based classification of DEGs. **(D)** The top 20 enriched signal pathways of DEGs. The circle size represents the number of genes and the color signifies *P*-value.

GO was applied for functional analysis of the 8,042 DEGs (Supplementary Table 4). In total, 847 terms were enriched in cellular component, of which 24 terms were significantly enriched, such as “membrane,” “membrane part,” and “cell periphery” (Figure 5A). Overall, 7,014 biological process terms were enriched, 71 of them to a significant extent (*P* < 0.05), including “cell communication,” “response to stimulus,” and “multicellular organismal process” (Figure 5B). Among the 1,903 terms enriched in molecular function, 32 terms were significantly enriched (*P* < 0.05), including “substrate-specific transporter activity,” “insulin receptor binding,” and “protein kinase A regulatory subunit binding” (Figure 5C).

The KEGG pathways with *P* < 0.05 were considered significantly enriched (Supplementary Table 4). In total, 256 pathways were enriched, among which 134 were significantly enriched (*P* < 0.05). The top 20 pathways, including “MAPK signaling,” “insulin signaling,” “Jak-STAT signaling,” and “phospatidylinositol signaling,” are listed in Figure 5D. We identified 245 and 187 DEGs enriched in the MAPK and insulin signaling pathways, respectively, indicating potential roles of these mechanisms in tail/rump fat metabolism of Altay and XFW sheep.

### GO and KEGG Analyses of DEGs Related to Adipose Metabolism

**FIGURE 6:**
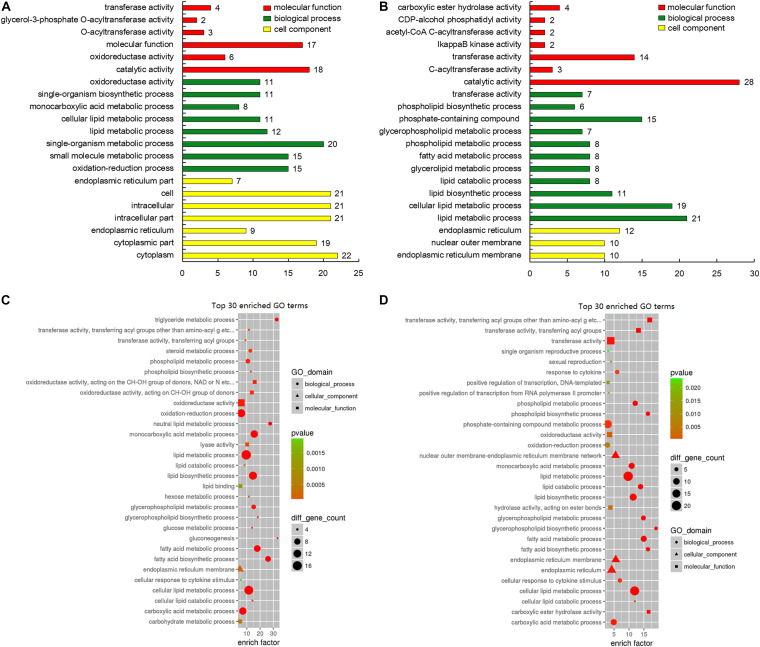
Gene ontology (GO) analysis of genes involved in lipid deposition-related regulation. **(A)** GO classification of upregulated genes (red, molecular function; green, biological process; yellow, cell component). **(B)** GO classification of downregulated genes (red, molecular function; green, biological process; yellow, cell component). **(C)** GO enrichment of the top 30 lipid metabolism-related upregulated genes. The circle size represents the number of genes involved in “biological process,” triangle size represents the number of genes involved in “cell component,” and square size represents the number of genes involved in “molecular function.” The color represents the *P*-value. **(D)** GO enrichment of top 30 lipid metabolism-related downregulated genes. The circle size represents the number of genes involved in biological process, triangle size represents the number of genes involved in cell component, and square size represents the number of genes involved in molecular function. The color signifies *P*-value.

GO analysis of the 198 DEGs disclosed significant enrichment of the molecular function terms such as “catalytic activity,” “molecular function,” and “transferase activity.” With regard to biological process terms, the processes, which related to adipose deposition and metabolism, were significantly enriched such as “lipid metabolic process,” “small molecule metabolic process,” “oxidation-reduction process,” and “single-organism metabolic process.” Among cellular component terms, such as the “cytoplasm,” “cytoplasmic part,” and “intracellular part” were significantly enriched (Figures 6A,B). The top 30 GO enrichment terms of DEGs are presented in Figures 6C,D.

To identify the biological pathways underlying adipose deposition, the 198 DEGs were mapped to the KEGG pathway database. The pathways with *P* < 0.05 were considered significantly enriched. Several pathways related to lipid metabolism were identified (Table 2).

**TABLE 2:** Lipid metabolism-related differentially expressed genes (DEGs) and enriched signaling pathways.

Pathway	DEGs	
	Upregulated	Downregulated
Ether lipid metabolism	*AGPS*, *PPAP2*, *SPLA2*, *CEPT1*, *PLD1*, *PLD2*	*ENPP2*, *PAFAH2*, *EPT1*, *PLA2G12A*, *PAFAH1B2*, *PAFAH1B1*, *MAPKBP1*
Sphingolipid metabolism	*GLA*, *SPTLC2*, *SPTLC3*, *SGMS*, *PPAP2*, *GLB1*, *KDSR*, *SGMS2*, *UGCG*, *SGPL1*	*CERK*, *ACER2*, *SGPP1*, *GALC*, *SPT*, *SPTLC1*, *ASAH1*, *SPHK1*, *DPL1*, *LACZASAH1*, *CER*, *ACER12*, *B4GALT6*
Alpha-linolenic acid metabolism	*ACOX1*, *BDHAB*, *CYP1A2*, *COX1*, *SPLA2*	*PLA2G12A*, *FADS2*, *FADA*, *FADIA*
Linoleic acid metabolism	*PTGS1*, *BDHAB*	*PLA2G12A*, *MAPKBP1*, *CYP2J2*, *CYP2C40*, *CYP2E1*
Arachidonic acid metabolism	*PTGS2*, *ATS2*, *EPHX2*, *COX1*, *CBR3*, *CBR1*	*CYP2U1*, *PLA2G12A*, *CYP4F3*, *GGT5*, *LTC4S*, *PTGES*, *CYP4A11*, *K15717*, *ALDH3A2*
Glycerolipid metabolism	*LPIN1*, *PLSC*, *LCLAT1*, *ALDH9A1*, *PPAP*, *GPAT12*, *SHROOM4*, *GLA*, *DGAT2*	*DGKA*, *DGKD*, *ALDH3A2*, *DGKH*, *AGPAT1*, *DGKQ*, *MOGAT3*, *GPAT4*, *GPAT3*, *ADH*, *LIP*, *ATS2*, *ACT2*, *DGAT1*
Biosynthesis of unsaturated fatty acids	*ACNAT*, *ELOVL5*, *ACOX1*	*ACOT7*, *FADS2*, *TECR*, *ACAA1*, *BAAT*, *RPB1*, *PTPLB*
Steroid hormone biosynthesis	*PGFS*, *UGT2B7*	*CYP7B1*, *CYP1B1*, *CYP1A1*, *AKR1C2*, *AKR1C4*, *AKR1C*, *HSD11B1*, *HSD11B2*,
Steroid biosynthesis	*SOAT1*, *ACNAT2*, *ACOT8*, *PODNL1*, *TM7SF2*	*CYP2R1*, *SC5DL*, *AKR1C4*, *HSD17B4*, *SCP2*, *ERG24*, *LBR*, *ERG3*
MAPK signaling pathway	*AKT2*, *AKT3*	*PLA2G12A*, *TNFRSF1A*, *CHUK*, *IKBKB*, *MAPK8*
Fatty acid degradation	*FADA*, *HELZ*, *ADIPOQ*, *C5ORF25*, *MFSD4*, *CPT*, *ACOX1*, *FADD*, *ACOX3*, *ACSL1*, *ACSL3*, *ACSL4*, *ACSL6*	*CPT1A*, *ALDH3A2*, *ACADL*, *ACAT1*, *HADHB*, *ECHS1*, *CYP4A11*, *ACAA1*, *PAAF*, *ECHDC3*, *HADHA*
Fatty acid elongation	*ELOVL5*, *C5ORF25*	*HADHB*, *ECHS1*, *ACAA2*, *SMR3A*, *HELZ*, *PTPLB*, *HACD*, *TECR*, *ACOT7*
Fatty acid biosynthesis	*FASN*, *ACACA*, *CBR4*	*TNS*, *TENC1*
Fat digestion and absorption	*ABCA1*, *DGAT2*, *AGPAT1*, *LPPR3*	*CD36*, *APOA1*, *PLA2G12A*, *SCARB1*, *MOGAT3*, *MAPKBP1*, *GOT2*, *CALB2*, *LPAAT*
Adipocytokine signaling pathway	*AKT2*, *AKT3*, *ACSL1*, *ACSL3*, *ACSL4*, *ACSL6*, *ACACA*, *ACACB*, *CD36*, *SLC27A2*, *FADD*, *G6PC*, *RXRB*, *OPTN*	*MAPK8*, *PRKAB1*, *PRKAB2*, *IRS2,CPT1A*, *ADIPOR2*, *ADIPOQ*, *PPARA*, *PPCK1*, *C1QTNF9*, *RXRA*, *PTPN11*, *LEP*, *CAMKK2*, *JAK2*, *STAT3*, *TNFRSF1A*, *TNFRSF1B*, *CHUK*, *IKBKB*, *MTOR*, *SLC2A4*, *NFKBIA*, *NFKBIB*, *ACSBG2*, *PCK2*, *PEPCK*, *STK11*, *C1QTNF1*, *PRKAG2*
PPAR signaling pathway	*FABP3*, *FABP4*, *SLC27A2*, *SLC27A6*, *ACSL1*, *ACSL3*, *ACSL4*, *ACSL6*, *PLIN1*, *ANGPTL4*, *SORBS1*, *PDPK*	*ADIPOQ*, *ACADL*, *AQP7*, *APOA1*, *SCP2*, *APM-1*, *ACAA1*, *CPT1A*, *ACOX1*, *ACOX2*, *C1QTNF1*, *CYP4A11*, *FADS2*, *PPCK1*, *PMP2*, *RXRA*, *RXRB*, *DBI*, *C1QTNF9*, *TRIM56*
Insulin signaling pathway	*ACACA*, *SORBS1*, *FASN*, *AKT2*, *AKT3*, *G6PC*, *PRKAA1*, *PRKAB2*	*MAPK8*, *PPCK1*, *IRS4*, *IKBKB*, *PDPK1*, *PRKAG2*, *SLC2A4*, *MTOR*
Metabolic pathways	*FASN*, *LPIN1*, *CDS2*, *PLD1*, *AGPS*, *PLD2*, *UGCG*, *SGMS2*, *AGPAT9*, *G6PC*, *DGAT2*, *GPAM*, *SGPL1*, *AGPAT6*, *TM7SF2*, *CBR3*, *GLB1*, *DGKE*, *SPTLC2*, *SPTLC3*, *ACOX1*, *PTGS2*, *EPT1*, *ETNK1*, *ACACA*, *ACSL1*, *ACSL3*, *ACSL4*, *ACSL6*	*PGS1*, *ACADL*, *IRS2*, *CYP2U1*, *PPCK1*, *ALDH3A2*, *PAFAH2*, *ACAT1*, *CYP2R1*, *HADHB*, *PLA2G12A*, *PAFAH1B2*, *CYP4F3*, *PCYT1A*, *HSD11B1*, *KDSR*, *SC5DL*, *PAFAH1B1*, *BDH1*, *AGPAT1*, *GGT5*, *DGKQ*, *LTC4S*, *ASAH1*, *AKR1A1*, *ECHS1*, *HSD11B2*, *CYP1A1*, *UGT2B*, *SPHK1*, *CYP4A11*, *ACAA1*, *DGKA*, *DGKD*, *ACER2*, *GALC*, *DGKH*, *SPTLC1*, *PTGES*
Glycerophospholipid metabolism	*EPT1*, *CDS2*, *PLD1*, *GPD1L*, *AGPAT9*, *PLD2*, *GPD2*, *AGPAT6*, *ETNK1*, *LCLAT1*, *GPAM*, *DGKE*	*PGS1*, *DGKA*, *DGKD*, *PLA2G12A*, *LPGAT1*, *PCYT1A*, *LYPLA1*, *PCYT2*, *GPD1*, *DGKH*, *DGKQ*, *AGPAT1*

In total, 32 DEGs were enriched in the PPAR signaling pathway, including 12 upregulated and 20 downregulated genes. Among these, 10 genes (*FABP3*, *FABP4*, *SLC27A2*, *SLC27A6*, *ACSL1*, *ACSL3*, *ACSL4*, *ACSL6*, *ANGPTL4*, and *PLIN1*) were highly expressed and 16 (*ADIPOQ*, *ACAA1*, *ACADL*, *AQP7*, *APOA1*, *SCP2*, *APM-1*, *CPT1A*, *ACOX1*, *ACOX2*, *C1QTNF1*, *CYP4A11*, *FADS2*, *PPCK1*, *RXRA*, and *PMP2*) were significantly downregulated in rump fat of Altay sheep (*P* < 0.05).

Furthermore, 44 DEGs were enriched in the adipocytokine signaling pathway, including 14 upregulated and 30 downregulated genes. Ten of these genes (*AKT2*, *AKT3*, *ACSL1*, *ACSL3*, *ACSL4*, *ACSL6*, *ACACA*, *ACACB*, *CD36*, and *SLC27A*) were significantly upregulated (*P* < 0.05) and 11 genes (*MAPK8*, *PRKAB1*, *PRKAB2*, *IRS2*, *CPT1A*, *ADIPOR2*, *ADIPOQ*, *PPARA*, *PPCK1*, *C1QTNF9*, and *RXRA*) were significantly downregulated in rump fat of Altay sheep (*P* < 0.05).

**FIGURE 7:**
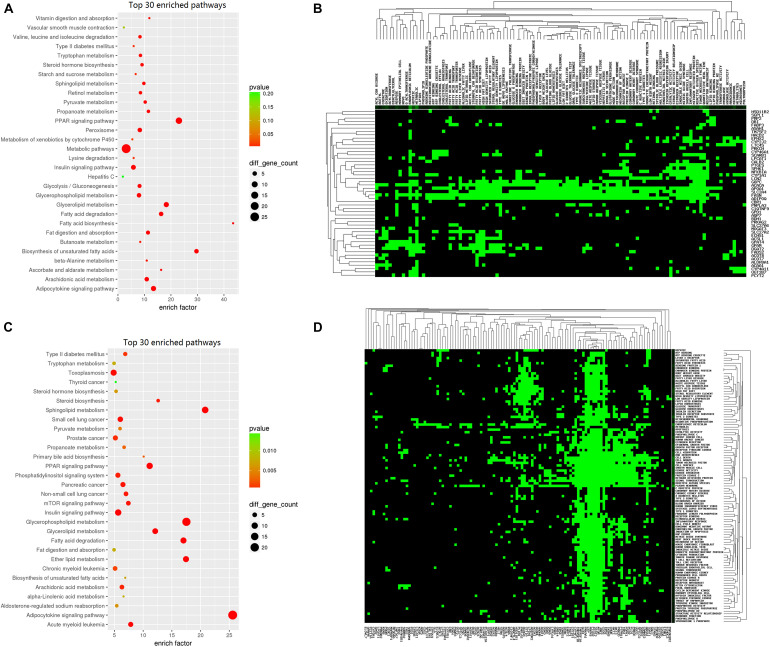
Kyoto Encyclopedia of Genes and Genomes (KEGG) analysis of lipid deposition-related regulation genes. **(A)** KEGG enrichment of the top 30 lipid metabolism-related upregulated genes. The circle size represents the number of genes and the color signifies the *P*-value. **(B)** Heatmap of lipid metabolism-related upregulated genes. Green color represents enriched genes. **(C)** KEGG enrichment of the top 30 lipid metabolism-related downregulated genes. The circle size represents the number of genes and the color signifies the *P*-value. **(D)** Heatmap of lipid metabolism-related downregulated genes. Green color represents enriched genes.

Other pathways, including “metabolic pathway,” “insulin signaling pathway,” and “glycerolipid metabolism,” additionally play important roles in fat metabolism. The top 30 enriched pathways are presented in Figures 7A,C. Heatmaps were generated and clearly depicted significantly enriched pathways and DEGs (Figures 7B,D) (*P* < 0.05).

Since the majority of the 198 DEGs were enriched in key pathways significantly related to fat metabolism, we proposed that these genes are potentially critical for sheep tail phenotype regulation and required to be further investigated.

### Interaction Network Analysis of Proteins Encoded by DEGs Related to Adipose Metabolism

**FIGURE 8:**
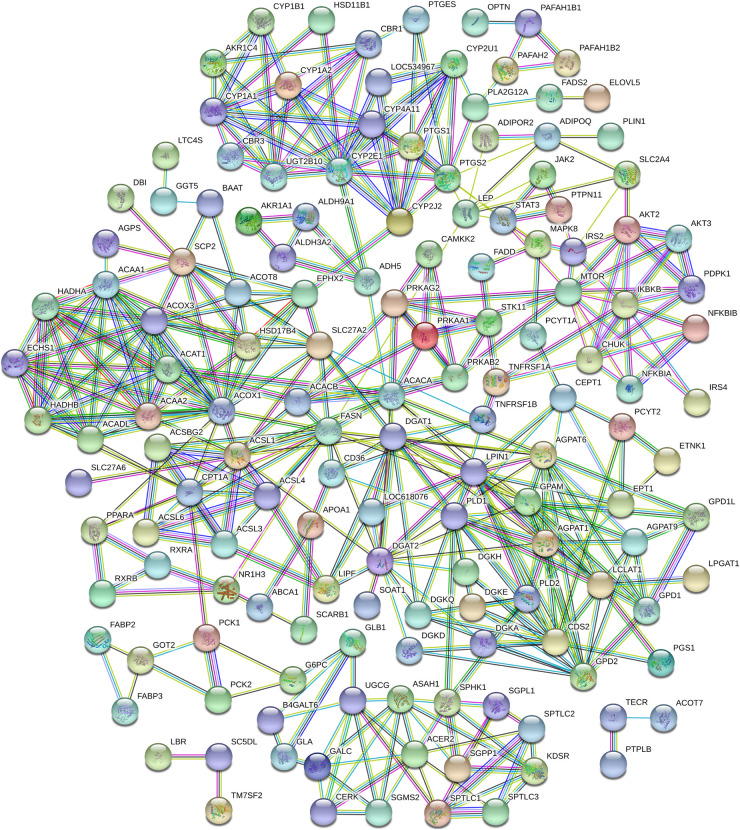
Protein-protein interaction analysis of 198 lipid metabolism-related differentially expressed genes (DEGs).

With the aid of STRING 11.0 software, an interaction network of proteins encoded by the 198 DEGs related to adipose metabolism was constructed (Figure 8), resulting in the detection of existing interactions among 148 genes. KEGG data disclosed 94, 37, 31, 26, 21, 25, and 19 interacting proteins related to the terms metabolic pathway (FDR ≤ 2.67e^–61^), “adipocytokine signaling pathway” (FDR ≤ 1.07e^–47^), “PPAR signaling pathway” (FDR ≤ 9.24e^–38^), “glycerophospholipid metabolism” (FDR ≤ 5.14e^–27^), fatty acid metabolism” (FDR ≤ 2.17e^–25^), “insulin resistance” (FDR ≤ 9.38e^–25^), and “fatty acid degradation” (FDR ≤ 1.43e^–23^), respectively. In view of the important roles of these pathways in adipose metabolism, we speculated that they may also influence the tail phenotypes of different sheep breeds by regulating fat metabolism in tail tissue.

**FIGURE 9:**
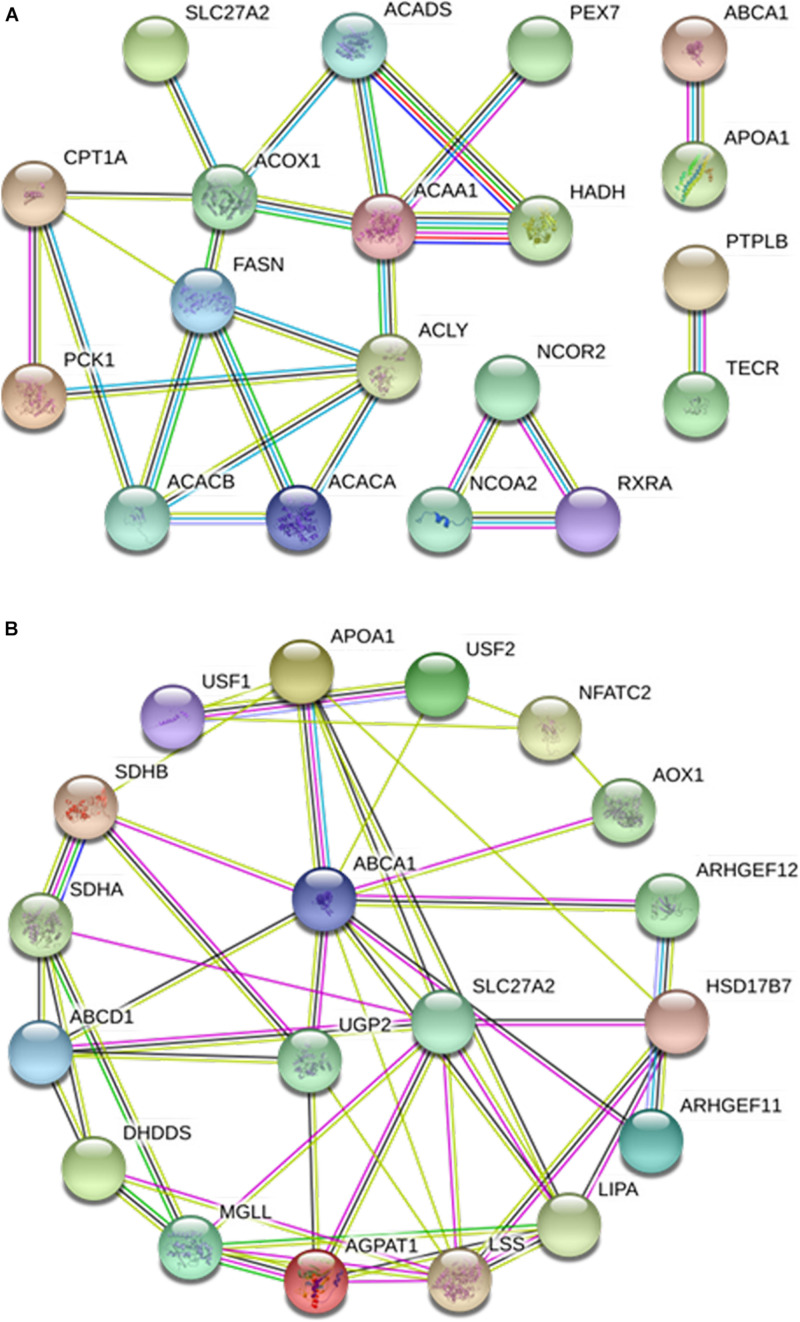
Protein-protein interaction analyses of lipid metabolism-related candidate proteins. **(A)** Interactions of 19 lipid metabolism-related candidate functional proteins. **(B)** Interactions of protein SLC27A2 and ABCA1.

The interaction network of 22 proteins related to fat deposition was further analyzed, which revealed interactions among 19 of the proteins (Figure 9A). The core nodes were identified as ACOX1, FASN, and ACAA1. ACOX1 interacted with ACADS, SLC27A2, CPT1A, FASN, and ACAA1. Interactions of FASN with CPT1A, ACACB, ACACA, ACLY, and ACOX1 were detected. ACAA1 showed interactions with PEX7, HADH, ACADS, ACOX1, and ACLY. GO analysis revealed that the majority of these proteins were related to the PPAR signaling pathway, fatty acid metabolism, and fatty acid biosynthetic process.

To further clarify the functions of *ABCA1* and *SLC27A2*, their interaction networks with other proteins were analyzed (Figure 9B). ABCA1 showed interactions with 10 proteins, including APOA1, UGP2, ARHGEF12, AOX1, and ARHGEF11, while SLC27A2 interacted with eight proteins, including ABCD1, AGPAT1, and HSD17B7.

### Detection of Variants in Candidate Genes Related to Tail Fat Metabolism

A total of 41,724 and 42,193 SNPs were detected in tail fat tissue transcriptomes of Altay and XFW sheep, respectively (Supplementary Tables 5,6). We specifically focused on the 22 candidate genes related to tail fat metabolism, which led to the identification of 13 SNPs in the coding regions of nine genes, among which 12 induced amino acid alterations (Table 3). The distribution of seven SNPs that induced amino acid substitutions were further investigated in Altay, XFW, and Hu sheep populations with different tail phenotypes (Table 4).

**TABLE 3:** The SNPs in candidate genes related to tail fat metabolism.

Gene	Position^a^	Basic	FR-chr base	FR-chr reads	TT-chr base	TT-chr reads	Style of amino acid mutation	Chromosome
*ACACA*	13081041	T	T	255	C;T	216;39	Glu-Lys	11
	13028657	A	G	255	A;G	226;28	Leu-Pro	11
*PPCK1*	57902435	C	C	94	TC	235;20	Glu-Lys	13
*ABCA1*	18100859	G	T;G	27;16	G	4	Pro-Leu	2
	18167532	G	G	166	A;G	48;14	Lys-Glu	2
*SLC27A2*	57036072	C	C	2	A;C	13;5	Met-Ile	7
*CPT1A*	45468209	T	T;G	13;11	G	4	Ser-Arg	21
	45468249	G	A;G	13;12	G	1	Ser-Ser	21
*FBP2*	31747535	G	A	4	G;A	27;7	Ile-Val	2
*FADS2*	31762518	C	C	2	T;C	37;16	Arg-Gly	21
	39768783	C	C	11	T;C	29;15	Arg-Trp	21
	39774594	A	G	20	G;A	35;31	Arg-Gly	21
*PLIN1*	20197576	C	C;G	251;2	T;C	189;64	Ala-Tyr	18

**FR*, fat rump; *TT*, thin tail; *chr* (in “chr base” and “chr reads”), chromosome. ^*a*^The location of the genetic variation in sheep genome (ISGC Oar_v3.1/oviAri3, August 2012).*

**TABLE 4:** Distribution of seven SNPs in three different sheep breed populations.

Gene SNP	Sheep breed	Genotype frequencies			Allele frequencies		Ratio	χ^2^
*ABCA1* 18100859		AA	AG	GG	A	G	A/G	
	Altay sheep (104)	0.327 (34)	0.481 (50)	0.192 (20)	0.567 (118)	0.433 (90)	1.311	0.045
	XFW sheep (104)	0.212 (22)	0.423 (44)	0.365 (38)	0.423 (88)	0.577 (120)	0.733	1.849
	Hu sheep (104)	0.135 (14)	0.365 (38)	0.500 (52)	0.308 (66)	0.683 (142)	0.464	2.552

***ABCA1* 18167532**		**TT**	**TC**	**CC**	**T**	**C**	**T/C**	

	Altay sheep (104)	0 (0)	0.106 (11)	0.894 (93)	0.053 (11)	0.947 (197)	0.056	0.324
	XFW sheep (104)	0.865 (90)	0.096 (10)	0.038 (4)	0.913 (190)	0.087 (18)	10.556	15.97**
	Hu sheep (104)	0 (0)	0.962 (100)	0.038 (4)	0.481 (100)	0.519 (108)	0.926	89.16**

***CPT1A* 45468209**		**GG**	**GT**	**TT**	**G**	**T**	**G/T**	

	Altay sheep (104)	0.346 (36)	0.654 (68)	0 (0)	0.673 (140)	0.327 (68)	2.058	24.54**
	XFW sheep (104)	0.385 (40)	0.577 (60)	0.038 (4)	0.673 (140)	0.327 (68)	2.058	10.05**
	Hu sheep (104)	0.173 (18)	0.644 (67)	0.183 (19)	0.495 (103)	0.505 (105)	0.981	8.661*

***FADS2* 39768783**		**CC**	**CT**	**TT**	**C**	**T**	**C/T**	

	Altay sheep (104)	0.106 (11)	0.894 (93)	0 (0)	0.553 (115)	0.447 (93)	1.237	68.01**
	XFW sheep (104)	0.385 (40)	0.615 (64)	0 (0)	0.692 (144)	0.308 (64)	2.250	20.54**
	Hu sheep (104)	0.683 (71)	0.317 (33)	0 (0)	0.803 (175)	0.139 (33)	5.303	3.698

***FBP2* 31747535**		**AA**	**AG**	**GG**	**A**	**G**	**A/G**	

	Altay sheep (104)	0.529 (55)	0.385 (40)	0.087 (9)	0.721 (150)	0.279 (58)	2.584	0.198
	XFW sheep (104)	0.644 (67)	0.269 (28)	0.087 (9)	0.779 (162)	0.221 (46)	3.522	4.964
	Hu sheep (104)	0.596 (62)	0.337 (35)	0.067 (7)	0.764 (159)	0.236 (49)	3.244	0.447

***PLIN1* 20197576**		**CC**	**CG**	**GG**	**C**	**G**	**C/G**	

	Altay sheep (78)	0 (0)	0.231 (24)	0.770 (80)	0.115 (24)	0.885 (184)	0.130	1.327
	XFW sheep (78)	0 (0)	0.077 (8)	0.923 (96)	0.038 (8)	0.962 (200)	0.040	0.125
	Hu sheep (78)	0 (0)	0.295 (30)	0.705 (74)	0.147 (30)	0.853 (178)	0.168	2.333

***SLC27A2* 57036072**		**GG**	**GT**	**TT**	**G**	**T**	**G/T**	

	Altay sheep (104)	0.337 (35)	0.587 (61)	0.077 (8)	0.630 (131)	0.370 (77)	1.701	6.915*
	XFW sheep (104)	0.038 (4)	0.125 (13)	0.837 (87)	0.101 (21)	0.899 (187)	0.112	89.163**
	Hu sheep (104)	0.385 (40)	0.596 (62)	0.019 (2)	0.683 (142)	0.317 (66)	2.151	14.70**

XFW, Xinjiang fine wool sheep. *P < 0.05 (χ^2^_0_._05_ = 5.99); **P < 0.01 (χ^2^_0_._01_ = 9.21).

Based on data obtained from 104 individuals of each sheep breed, the distribution of g.18167532T/C (Oar_v3.1) mutation of *ABCA1* and g.57036072G/T (Oar_v3.1) mutation of *SLC27A2* in these three populations showed significant differences. For the g.18167532T/C mutation of *ABCA1*, 89.4% individuals in the fat-rumped Altay sheep population were CC genotype, 96.2% of fat-tailed Hu sheep (a breed with a short fat-tailed phenotype between Altay and XFW sheep) were TC genotype, and 86.5% individuals in the long thin-tailed XFW group were TT genotype. The results of the Chi-square test showed that this SNP was not in Hardy-Weinberg equilibrium in XFW and Hu sheep populations (*P* < 0.01) while the Altay population was in Hardy-Weinberg equilibrium at this site (*P* > 0.05).

For the g.57036072G/T mutation of *SLC27A2*, G allele was main genotype in fat-rumped Altay sheep and short fat-tailed Hu sheep populations (63.0 and 68.3% of individuals had the G allele, respectively) while in the thin-tailed XFW sheep population, 89.9% of individuals had the T allele. The three sheep populations were not in Hardy-Weinberg equilibrium at this SNP.

Due to the *ABCA1* and *SLC27A2* were extremely expressed in tail fat tissues of Altay and XFW sheep, and the distribution of mutations in their gene sequences were significantly different in sheep breeds with different tail phenotype, we hypothesized that they might play potential roles in influencing the tail phenotype in sheep breeds and required to be further investigated.

## Discussion

### Transcriptome Studies of Tail/Rump Fat From Sheep

Tail/rump fat deposition in sheep breeds, which distribute in high-latitude regions, facilitate their adaptation to harsh environments. However, these fat-tailed/rumped sheep are undesirable for many modern sheep industry and consumers. Uncovering the mechanisms underlying tail fat deposition would aid in not only improving the meat quality of existing breeds but also selecting the lean-tailed sheep breeds. Meanwhile, the developments in RNA-Seq have facilitated elucidation of the regulatory mechanisms underlying a single trait.

Transcriptome and DEGs of tail fat tissue of sheep breeds with different tail phenotypes have been investigated in recent years (Wang et al., 2014; Miao et al., 2015; Li et al., 2018; Ma et al., 2018), leading to the identification of several important genes and pathways related to fat deposition. However, since the sheep breeds selected for these studies have not always been suitable, the results may not comprehensively reflect differences in the molecular mechanisms regulating tail fat deposition. Guangling large-tailed sheep, Lanzhou fat-tailed sheep, and small-tailed Han sheep studied by Li et al. (2018) and Ma et al. (2018) are fat-tailed breeds that display some differences in tail size. Their transcriptomes are highly similar, and thus limited DEGs have been identified among these breeds. Furthermore, fat-tailed sheep breeds investigated in these earlier studies are distributed in temperate rural regions of China where the winter is not extremely cold and feed is sufficient over the whole year. Consequently, the speed of deposition and mobilization of tail fat in these breeds may be slower, compared to the breeds in high-latitude areas.

Here, we used two highly suitable sheep breeds, fat-rumped Altay and thin-tailed XFW sheep, for investigating transcriptome differences in tail fat tissue. These two breeds were selected for several reasons. Firstly, both groups of sheep are traditionally localized in the Xinjiang province of China, which avoids the potential effects of distinct environment factors on transcriptome data. Secondly, the tail types are extremely different. Thus, we speculated that there may be significant differences between their tail fat transcriptomes. Thirdly, the living environment of Altay sheep is characterized by long and cold winter and relatively short summer and autumn seasons. The average temperature across the whole year is about 0.7°C to 4.9°C and can drop as low as -51.5°C in winter. To adapt the extreme climate, Altay sheep get the ability to rapidly deposit massive amounts of fat in the rump by consuming sufficient nutritious grass during the warmer seasons and rapidly mobilize in extreme winter conditions to obtain energy and sustain life. Owing to this ability, Altay sheep can live outdoors, even at extreme temperatures of –30 to –40°C. Thus, the two sheep breeds represent good models to compare transcriptome and DEGs profiles that regulate tail fat deposition.

Using RNA-Seq, a total of 19,878 genes were identified in tail fat tissues, among which 8,042 were differentially expressed between the two sheep breeds (FDR ≤ 0.001 and | log_2_ratio| ≥ 1). Li et al. (2018) reported 5,395 DEGs between tail fat tissues of Guangling large-tailed sheep and small-tailed Han sheep. In addition, 646 DEGs between Kazak and Tibetan sheep were reported by Wang et al. (2014), 390 DEGs between Lanzhou fat-tailed and Tibetan sheep by Ma et al. (2018), and 602 DEGs between small-tailed Han and Dorset sheep by Miao et al. (2015). The numbers of total genes and DEGs identified in the current study were significantly higher than previously reported figures, which may be attributable to the higher suitability of our animal models.

Among the 3,965 DEGs with significantly different expression (FDR ≤ 0.001 and | log_2_ratio| ≥ 2), 707 were upregulated and 3,258 downregulated in rump fat tissue of Altay sheep. The total number of upregulated genes was significantly lower than that of downregulated genes, similar to the trend observed by the groups of Wang et al. (2014) and Miao et al. (2015). We speculated that the majority of downregulated genes may play important roles in tail fat metabolism in thin-tailed sheep breeds from as early as the time of domestication. Under both human-induced artificial and natural selection, sheep breeds with the powerful ability of efficient tail fat deposition evolved *via* upregulation of genes promoting fat deposition. Since these types of sheep were more adaptable to harsh environments, their numbers gradually increased and new breeds developed, such as Altay, Guangling large-tailed, Lanzhou fat-tailed, and other fat-tailed sheep breeds worldwide.

### DEGs in Sheep Tail/Rump Fat Tissue

DEGs in tail fat tissues of Altay and XFW sheep may play key roles in determining tail phenotype differences. To examine this hypothesis, we focused on the 198 DEGs showing significant differences in expression (FDR ≤ 0.001 and | log_2_ratio| ≥ 2) and closely related to fat metabolism. Among the KEGG pathways of these genes, 134 pathways, which related to adipose metabolism, were significantly enriched, including adipocytokine signaling, PPAR signaling, fat digestion and absorption, and glycerolipid metabolism.

The PPAR signaling pathway regulates cellular differentiation, energy balance, and lipid metabolism (Gross et al., 2017). PPAR exists as α, β, and γ isoforms (Wu et al., 1999), and the expression of PPARγ is necessary for adipocyte differentiation (Farmer, 2006; Floyd and Stephens, 2012). Activation of PPARγ is reported to be essential for deposition of intramuscular fat (Hausman et al., 2018). Previous research indicates that the majority of these upregulated genes are critical for fat deposition while downregulated genes are related to fat mobilization. In bovine mammary glands, mRNA abundance at 60 days postpartum of *FABP3* and *ACSL1* were 80- and 7-fold greater relative to 15 days antenatal, with peak expression of *SLC27A2* and *SLC27A6* at 240 and 15 days relative to parturition, respectively, which are significantly associated with milk fat synthesis (Bionaz and Loor, 2008). ANGPTL4 promotes LPL protein intracellular degradation and triglyceride levels in adipocytes (Dijk et al., 2016). PLIN1, an Fsp27 activator, interacts with the CIDE-N domain of Fsp27 and markedly enhances lipid droplet growth by promoting lipid exchange and transfer (Sun et al., 2013). ADIPOQ, an important adipocytokine, modulates glucose and fatty acid oxidation (Abbasi et al., 2004), and its gene sequence polymorphisms are associated with adipose deposition in pig and cattle (Dall’Olio et al., 2009; Choi et al., 2015). ACAA1 and ACADL play critical roles in beta-oxidation of fatty acids (Houten and Wanders, 2010).

*AKT*, an upregulated gene, has three isoforms in mammals (designated *AKT1*, *AKT2*, and *AKT3*) that are implicated in the regulation of widely divergent cellular processes, such as metabolism, differentiation, proliferation, and apoptosis (Lawlor and Alessi, 2001). In mouse adipocytes, upon rapid activation of AKT2, glucose transporter 4 (GLUT4) translocates to the cell surface and glucose transportation is accelerated (Ng et al., 2008). Most of the downregulated genes were negatively correlated with fat deposition. In adipose tissue of high fat diet-induced obese rats, *MAPK8* is significantly downregulated and apoptosis of adipocytes inhibited, which may be the main contributory factor to obesity (Qiu et al., 2010). Mutations of *PRKAB1* and *PRKAB2* are significantly associated with meat quality traits in pigs (Fontanesi et al., 2003).

Upon further analysis of these DEGs and their pathways, we observed involvement of a number of DEGs in multiple pathways. For instance, *ACSL1*, *ACSL3*, *ACSL4*, and *ACSL6* contribute to regulation of adipocytokine signaling, PPAR signaling, fatty acid degradation, and metabolic pathways. *MAPK8*, *AKT2*, and *AKT3* are involved in MAPK signaling, adipocytokine signaling, and insulin signaling pathway, while *FASN* and *ACACA* participate in regulation of fatty acid biosynthesis, insulin signaling, and metabolic pathways (Table 2). The mechanisms underlying fat metabolism are complex. Fat is not only a tissue used to store energy in animals but also an important endocrine tissue involved in regulating crucial physiological and biochemical reactions (Galic et al., 2010; Ohashi et al., 2011). Accordingly, fat metabolism is regulated by an elaborate network composed of numerous signaling pathways. We speculated that these genes involved in multiple pathways may play bridging roles to connect these signaling mechanisms.

Among the 22 candidate genes, *ABCA1*, *ACACA*, and *CIDEC* were significantly upregulated in rump fat tissue of Altay sheep (*P* < 0.01) with 5.37, 6.75, and 5.86 times higher expression, compared to tail fat tissue of XFW sheep. *C1QTNF1* and *HSL* were significantly downregulated in rump fat tissue of Altay sheep with 0.15 and 0.13 times expression relative to tail fat tissue of XFW sheep (*P* < 0.01). ABCA1 is a membrane transporter protein that plays an essential role in the efflux of cholesterol from peripheral tissues back to the liver for participating in lipid metabolism (Zhao et al., 2011). In sheep reared under intensive conditions and offered sufficient feed, *ACACA* in muscle was significantly upregulated and the fat deposition accelerated (Dervishi et al., 2011). CIDEC (FSP27) located on the surfaces of lipid droplets of adipocytes could promote enlargement or fusion of lipid droplets *via* clustering and lipid transfer (Li et al., 2017; Slayton et al., 2019; Price et al., 2020). Meanwhile, CIDEC (FSP27) suppressed HSL located on lipid droplet surfaces and inhibited lipolysis (Li et al., 2014). In Altay sheep fasted for 4 weeks, the *CIDEC* level in rump fat tissue was significantly downregulated (Gao et al., 2015). In human liver samples of individuals with obesity and diabetes mellitus, *CIDEC* was significantly upregulated (Keller et al., 2008; Karki, 2019). The collective studies confirmed essential roles of these candidate genes in fat metabolism.

In view of the significant differences in expression levels of these genes between tail fat tissues of Altay and XFW sheep, and their signaling pathways being closely related to fat metabolism, we speculated that the genes may be potential regulators of tail phenotype differences of sheep and need to be further investigated.

### Relevance of Gene Variants and Tail Fat Deposition of Sheep

Previous research has confirmed that a number of gene variants are closely related to tail phenotypes of sheep. Using Ovine SNP50k BeadChip, Moradi et al. (2012) investigated gene variants in two Iranian sheep breeds with fat tail and thin tail phenotypes, respectively. The group identified several mutations that were significantly different between the fat-tailed and thin-tailed sheep breeds in three regions located on chromosomes 5, 7, and X (Moradi et al., 2012). Our group additionally showed that polymorphisms of g.59571364, g.59912586, g.60149273, and g.59383635 loci on Chromosome X are markedly related to tail fat deposition ability of sheep breeds (Gan et al., 2013a, b; Zhang et al., 2013).

RNA-Seq offers novel opportunities for the efficient detection of transcriptome variants (SNPs and short insertion/deletions) in different tissues and species (Cánovas et al., 2010; Cox et al., 2012). Besides whole-genome sequencing, RNA-Seq offers a cost-effective approach for identifying variations and potentially causal mutations underlying the analyzed phenotypes (Hudson et al., 2012; Suárez-Vega et al., 2015). Using RNA-Seq, Suárez-Vega et al. (2017) detected 57,795 variants in the regions harboring quantitative trait loci (QTL) for mild yield, protein, and fat percentages in sheep, among which 21.44% were novel. In the current study, we detected 41,724 and 42,193 SNPs in tail fat tissue transcriptomes of Altay and XFW sheep, respectively, using RNA-Seq. We further focused on SNPs in 22 candidate genes related to tail fat metabolism, 12 of which altered the encoded amino acid (Table 3).

The distributions of g.18167532T/C mutation of *ABCA1* and g.57036072G/T mutation of *SLC27A2* were significantly different in the three sheep breed populations with distinct tail phenotypes (Table 4). *ABCA1* encodes a key protein regulating apolipoprotein-mediated efflux of cholesterol and phospholipid from peripheral cells to high-density lipoprotein-cholesterol (HDL-C) (Srivastava, 2002; Fredenrich and Bayer, 2003). Most previous studies have focused on the association of *ABCA1* gene polymorphisms with human disease. The SNPs rs4149267, rs1800977, rs1800978, and rs2230806 of *ABCA1* are associated with HDL-C concentrations in Caucasian, Sacramento, and French populations (Prochay et al., 2012; Clifford et al., 2013). A significant association was observed between the SNP of *ABCA1* and type 2 diabetes in patients of Han Chinese ancestry and Japanese population (Daimon et al., 2005; Kong et al., 2015). A rs2230806 genetic variation of *ABCA1* was significantly related to the development and severity of coronary artery disease (CAD) in an Iranian population. Moreover, the K allele of *ABCA1* R219K polymorphism has been shown to exert a protective effect against CAD risk and is correlated with decreased severity of CAD, independently of plasma lipid levels (Kolovou et al., 2011; Ghaznavi et al., 2018). Here, we detected a g.18167532T/C mutation of *ABCA1*. Its distribution in different sheep breeds was significantly related to tail fat deposition ability, which required to be further investigated.

The significance of mutations in *SLC27A* isoforms has been established in previous reports. Wang et al. (2007) identified a SNP in exon 7 leading to an amino acid alteration in Large White and Meishan pig breeds, which was significantly correlated with growth and carcass traits. SNPs at *SLC27A1* and *SLC27A4* were associated with saturated fatty acid and stearic acid contents in longissimus dorsi muscle of pig (Melo et al., 2013). Two SNPs in exon 3 and 3’UTR of bovine *SLC27A1* exerted effects on milk production traits, such as milk protein and milk fat percentages, in Chinese Holstein cattle (Lu et al., 2010). In the present investigation, the genotype of the g.57036072G/T mutation of *SLC27A2* was distinct in three sheep breeds with different tail phenotypes and the G allele was significantly related to fat rump phenotype in Altay sheep (Table 4).

The two mutations of *ABCA1* and *SLC27A2* identified in this study led to alterations in the encoded amino acids. In view of their significant association with tail phenotype of sheep, the issue of whether these mutations affect rump fat deposition in Altay sheep by influencing the functions of the corresponding proteins requires further investigation. Here, we confirmed relationships between limited SNPs and tail fat deposition traits of sheep. Further research is warranted to ascertain the associations of several other detected genes with tail fat metabolism.

## Conclusion

In this research, we examined the differences in transcriptome profiles and sequences of tail fat tissue from Altay and XFW sheep breeds that are distributed within the same province in China but display distinct tail fat deposition traits by applying RNA-Seq followed by qRT-PCR validation to confirm the reliability of our findings. DEGs were identified and their functions evaluated *via* GO and KEGG analyses. The genes associated with fat metabolism were filtered out for further analysis. Based on the data, 22 candidate genes and two SNPs were identified that potentially contribute to differences in tail fat deposition abilities of sheep. Determination of the specific roles of these DEGs and candidate genes in tail fat deposition ability may aid in the selection of lean-tailed sheep breeds.

## Data Availability Statement

All datasets generated for this study are included in the article/Supplementary Material. The raw reads data was submitted to the Short Read Archive (SRA) under the accession number SRR11586694-11586695 and BioProject accession number PRJNA627341 (https://dataview.ncbi.nlm.nih.gov/?search=SUB7313538).

## Ethics Statement

The animal study was reviewed and approved by the Biological Studies Animal Care and Use Committee, Xinjiang Production and Construction Corps, China, and the Ethics Committee of Xinjiang Academy of Agricultural and Reclamation Sciences.

## Author Contributions

WZ and SG conceived the study, conducted the data analysis, and prepared figures and tables. JW, MX, SW, and JY performed the sample collection and total RNA preparation. WZ, JW, and MX performed the qRT-PCR validation and SNP detection. WZ wrote the manuscript with the assistance of JW and SG. All authors read and approved the final manuscript.

## Conflict of Interest

The authors declare that the research was conducted in the absence of any commercial or financial relationships that could be construed as a potential conflict of interest.

## Footnotes

1 http://cole-trapnell-lab.github.io/cufflink/

2 http://www.geneontology.org/

3 http://www.kegg.jp/kegg/pathway.html

4 http://genome.ucsc.edu/

## Supplementary Material

Supplementary Figure 1 Length distribution of contigs and unigenes.

Supplementary Figure 2 Expression levels of 22 genes detected by RNA-Seq.

Supplementary Table 1 Primers used in qRT-PCR.

Supplementary Table 2 Primers used in detecting genetic variation of candidate genes related to the lipid metabolism.

Supplementary Table 3 All the annotated genes in the two libraries.

Supplementary Table 4 DEGs with the FDR value and the absolute value.

Supplementary Table 5 SNPs detected in Altay sheep.

Supplementary Table 6 SNPs detected in XFW sheep.
