# Supplementary material for: Comparative Transcriptome Analysis of Key Genes and Pathways Activated in Response to Fat Deposition in Two Sheep Breeds With Distinct Tail Phenotype
Source: Front Genet. 2021 Apr 8;12:639030. doi: 10.3389/fgene.2021.639030 (PMC8060577; doi:10.3389/fgene.2021.639030)
Supplement: Supplementary Figure 1 — Length distribution of contigs and unigenes. [file Data_Sheet_1.ZIP › Supplementary files/Figure S1 Length distribution of unigenes.pdf]

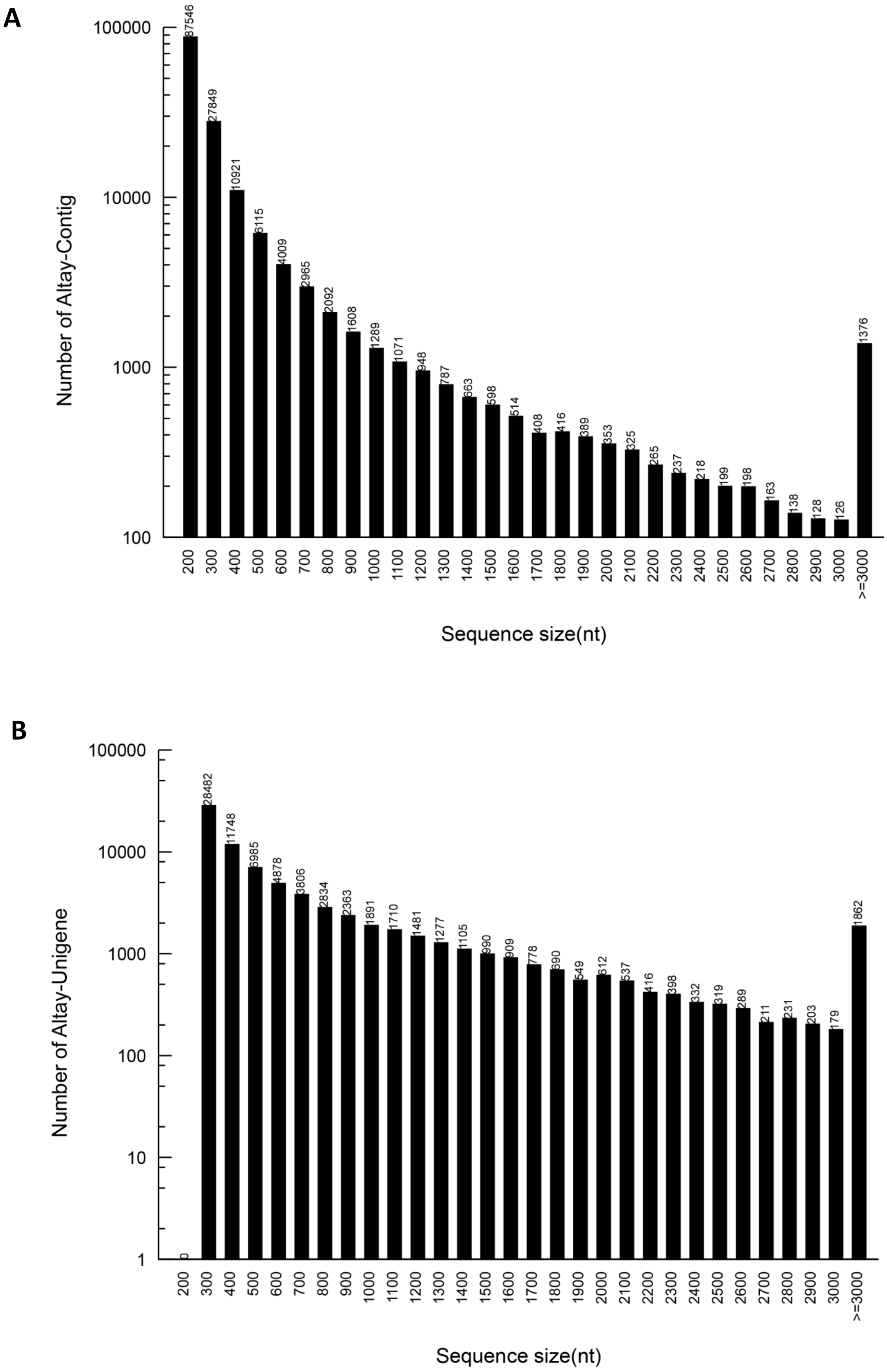
A
Number of Altay-Contig
100000
10000
1000
100
87546
27849
10921
6115
4009
2965
2092
1608
1289
1071
948
787
663
598
514
408
416
389
353
325
265
237
218
199
198
163
138
128
126
1376
200
300
400
500
600
700
800
900
1000
1100
1200
1300
1400
1500
1600
1700
1800
1900
2000
2100
2200
2300
2400
2500
2600
2700
2800
2900
3000
>=3000
Sequence size(nt)
B
Number of Altay-Unigene
100000
10000
1000
100
10
1
0
28482
11748
6985
4878
3806
2834
2363
1891
1710
1481
1277
1105
990
909
778
690
549
612
537
416
398
332
319
289
211
231
203
179
1862
200
300
400
500
600
700
800
900
1000
1100
1200
1300
1400
1500
1600
1700
1800
1900
2000
2100
2200
2300
2400
2500
2600
2700
2800
2900
3000
>=3000
Sequence size(nt)

**C**

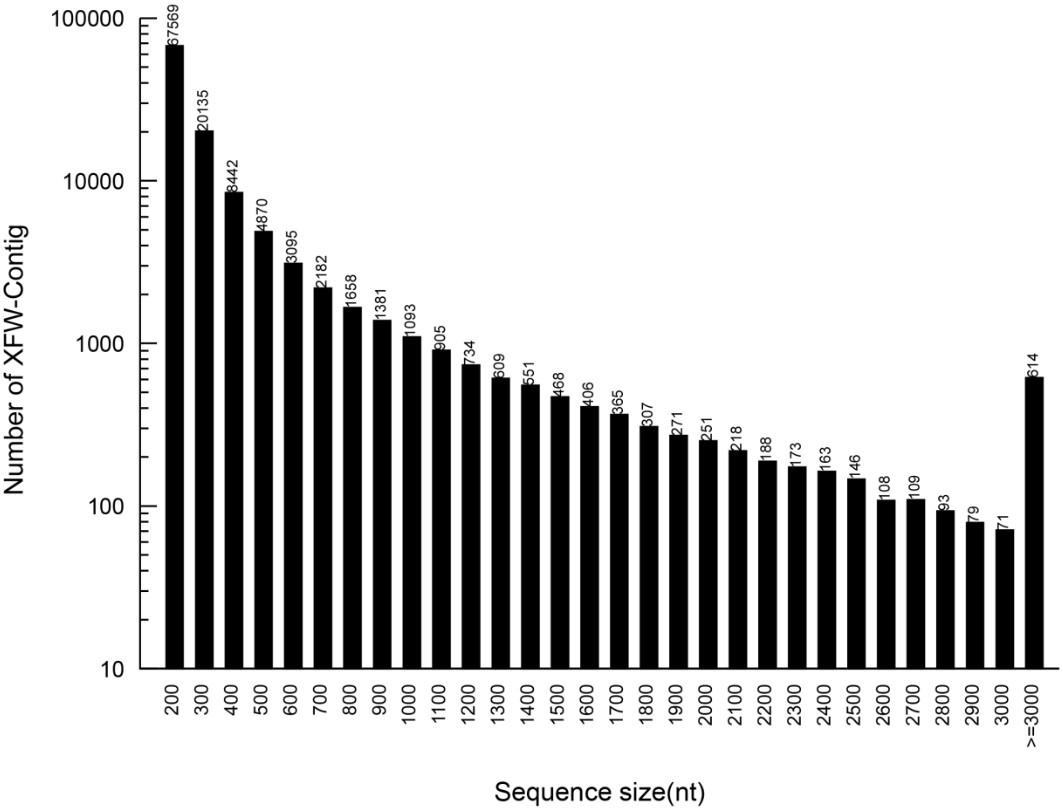


**D**

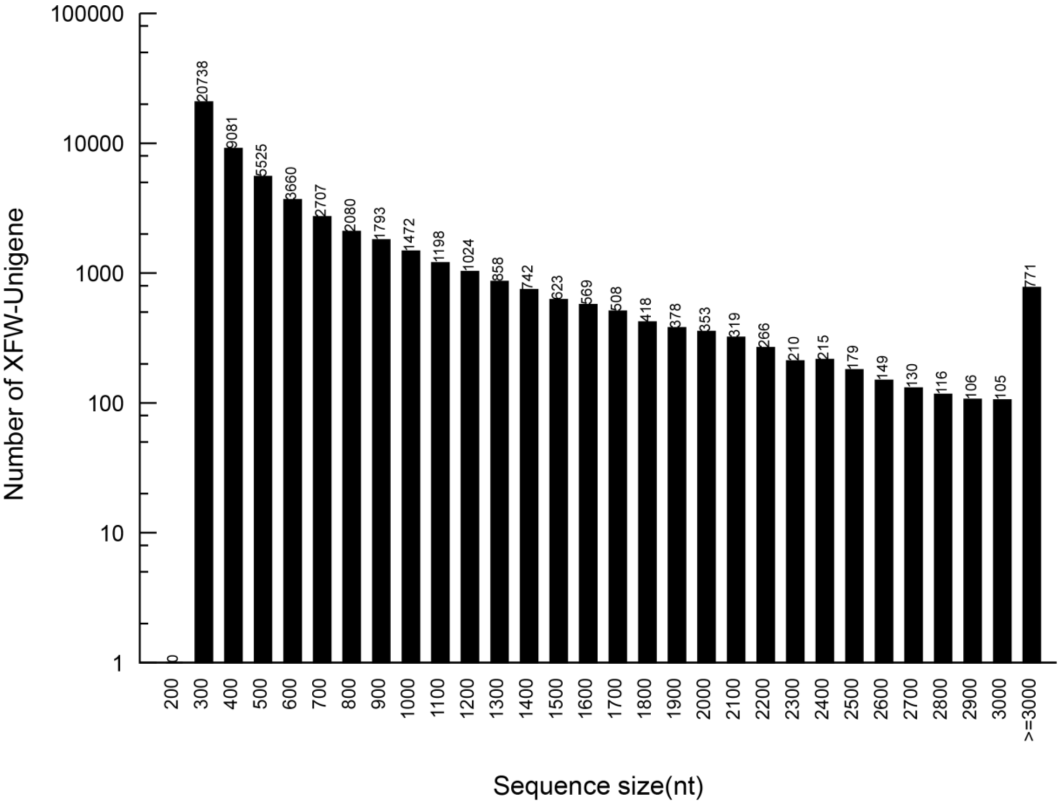


**Figure S1** **a** Length distribution of Altay contig; **b** Length distribution of Altay unigene; **c** Length distribution of XFW contig; **d** Length distribution of XFW unigene.
