# Supplementary material for: Comparative Transcriptome Analysis of Key Genes and Pathways Activated in Response to Fat Deposition in Two Sheep Breeds With Distinct Tail Phenotype
Source: Front Genet. 2021 Apr 8;12:639030. doi: 10.3389/fgene.2021.639030 (PMC8060577; doi:10.3389/fgene.2021.639030)
Supplement: Supplementary Figure 1 — Length distribution of contigs and unigenes. [file Data_Sheet_1.ZIP › Supplementary files/Table S2 Primers used in detecting SNP.docx]

**Table 2 Primers used in detecting genetic variation of the lipid metabolism-related candidate genes**

| **Gene** | **Primer sequence (5’-3’)** | **Size (bp)** |
| --- | --- | --- |
| *CPT1A*  45468209 | F: GAAGGAGATGTCACCAAAGTGC | 210 |
|  | R: CATAGCTGTCCATCGACGTTTC |  |
| *FBP2*  31747535 | F: GTGTGTGCTACTGACTTCTG | 201 |
|  | R: GGAAACTTCTTTTTCTGCAAAT |  |
| *PLIN1*  20197576 | F: GAACAGCATCAGTGTGCCCATTG | 196 |
|  | AGGGAGGAAGAACTCTACCATC |  |
| *FADS2*  39768783 | F: TGAGGAAGACTGCTGAGGACAT | 146 |
|  | R: CGTAATGATGGTTGGAATCCGG |  |
| *ABCA1*  18100859 | F: ACAACCTCTCTCTGCCATGG | 176 |
|  | R: ACCTCCTTCAGCACCATTAC |  |
| *ABCA1*  18167532 | F: AACCCTGACCTGAAGCCTGT | 251 |
|  | R: AGAGTCCTCACCCTGAAGAG |  |
| *SLC27A2*  57036072 | F: TCATCAGGAGATTTGGGGAT | 237 |
|  | R: GGTGATATAGATGGGTCATG |  |
