# Supplementary material for: Comparative Transcriptome Analysis of Key Genes and Pathways Activated in Response to Fat Deposition in Two Sheep Breeds With Distinct Tail Phenotype
Source: Front Genet. 2021 Apr 8;12:639030. doi: 10.3389/fgene.2021.639030 (PMC8060577; doi:10.3389/fgene.2021.639030)
Supplement: Supplementary Figure 1 — Length distribution of contigs and unigenes. [file Data_Sheet_1.ZIP › Supplementary files/Table S1 Primers used in qRT-PCR.docx]

**Table S1 Primers sequences for qRT-PCR of the lipid metabolism-related candidate functional genes**

| **Gene** | **Primer sequence (5’-3’)** | | **Size (bp)** |
| --- | --- | --- | --- |
| *ABCA1* | F: CTGATGTCAGTCTCCGCAAG | R: CCAAGAGCTGTTCAGCATGA | 303 |
| *ACADL* | F: AGATTTACAGACGGTGCAGC | R: GCAAAAGCTTATGTGGATGC | 240 |
| *ANGPTL4* | F: ACTTGGGACCAGGACCACGA | R: CACCTCCATCCTGGTACAGC | 214 |
| *CIQTNF1* | F: GAAGCCCCTACACAGCAACG | R: GAAGAACGCAGAGGAGGTGG | 197 |
| *CPT1A* | F: AACGGGATCCACGCCATCCT | R: CAGACACCATCCAGCACCTG | 165 |
| *ELOVL5* | F: CTTGCTAATCGTGTGGCTGG | R: AGTTAGTGACGGGAGTATGG | 139 |
| *IRS2* | F: ACGGCAAGCTGCTGCCCAAC | R: GTACGTGCTCATGAGCTCGC | 204 |
| *Lpin1* | F: GACCACGTCTTCCCTTTGCT | R: CTGCATCAGCCTAGAAGGTT | 141 |
| *PLIN1* | F: CAAGGATGCTGCCAAGGCAC | R: GCTACCAGCGAGTAACGTAT | 254 |
| *Rxra* | F: TTTGGGGGTCGCCTAAGGC | R: CTCTAGTGGACCGGAGGTCT | 198 |
| *SORBS1* | F: GCTGTTTCAAGTACCGACTC | R: CGGAAAGAAGAGTGGGAGAG | 219 |
| *ACAA1* | F: ATGTCCCTGGCTGACAGAGG | R: GTGCCTGTTACCACCACGGT | 233 |
| *ACACA* | F: GTTTGTGGAGGTGGAGGGAA | R: CCCTCAACGTAGGAAGAGCT | 297 |
| *CIQTNF9* | F: TAGAGGCCAATAATCCGCAG | R: CACTGTGGGAGCTTATGACT | 326 |
| *CYP4A11* | F: GCCTACATCCAGGCCATCAG | R: GAGCAGCTTGTCTGATGAGG | 283 |
| *FADS2* | F: TGCAACACGATTACGGCCAC | R: GCTGCATGTGTTTGTCCTGG | 192 |
| *FASN* | F: TCCTGAGAGACGCCATGCTG | R: GTGGTCTTCTCCTCGGTGAG | 148 |
| *FBP2* | F: TCGCAACATTGTGGCTGCTG | R: CGAGGGCTATGCCAAGTATT | 188 |
| *PMP2* | F: GTGGTCATAGACAAGGTGGC | R: GCACATTTCCCTAATCCCAG | 134 |
| *PPCK1* | F: AACCCCAATGCCATCAAGAC | R: GAGGGCATCATCTTTGGAGG | 275 |
| *PTPLB* | F: GCTCCTGGCAAACTCCTTAC | R: CAAGCACATTTCGTAGTGGC | 196 |
| *SLC27A2* | F: TCTATGCTTCCACTGAAGGC | R: GAACCTGGACTCCTGGTTTG | 199 |
| *GAPDH* | F:CTGACGTGCCGGCGTGGACA | R: GTAGAAGAGTGAGTGTCGCT | 149 |
