# Supplementary material for: Comparative Transcriptome Analysis of Key Genes and Pathways Activated in Response to Fat Deposition in Two Sheep Breeds With Distinct Tail Phenotype
Source: Front Genet. 2021 Apr 8;12:639030. doi: 10.3389/fgene.2021.639030 (PMC8060577; doi:10.3389/fgene.2021.639030)
Supplement: Supplementary Figure 1 — Length distribution of contigs and unigenes. [file Data_Sheet_1.ZIP › Supplementary files/Figure S2 Expression levels of 22 genes.pdf]

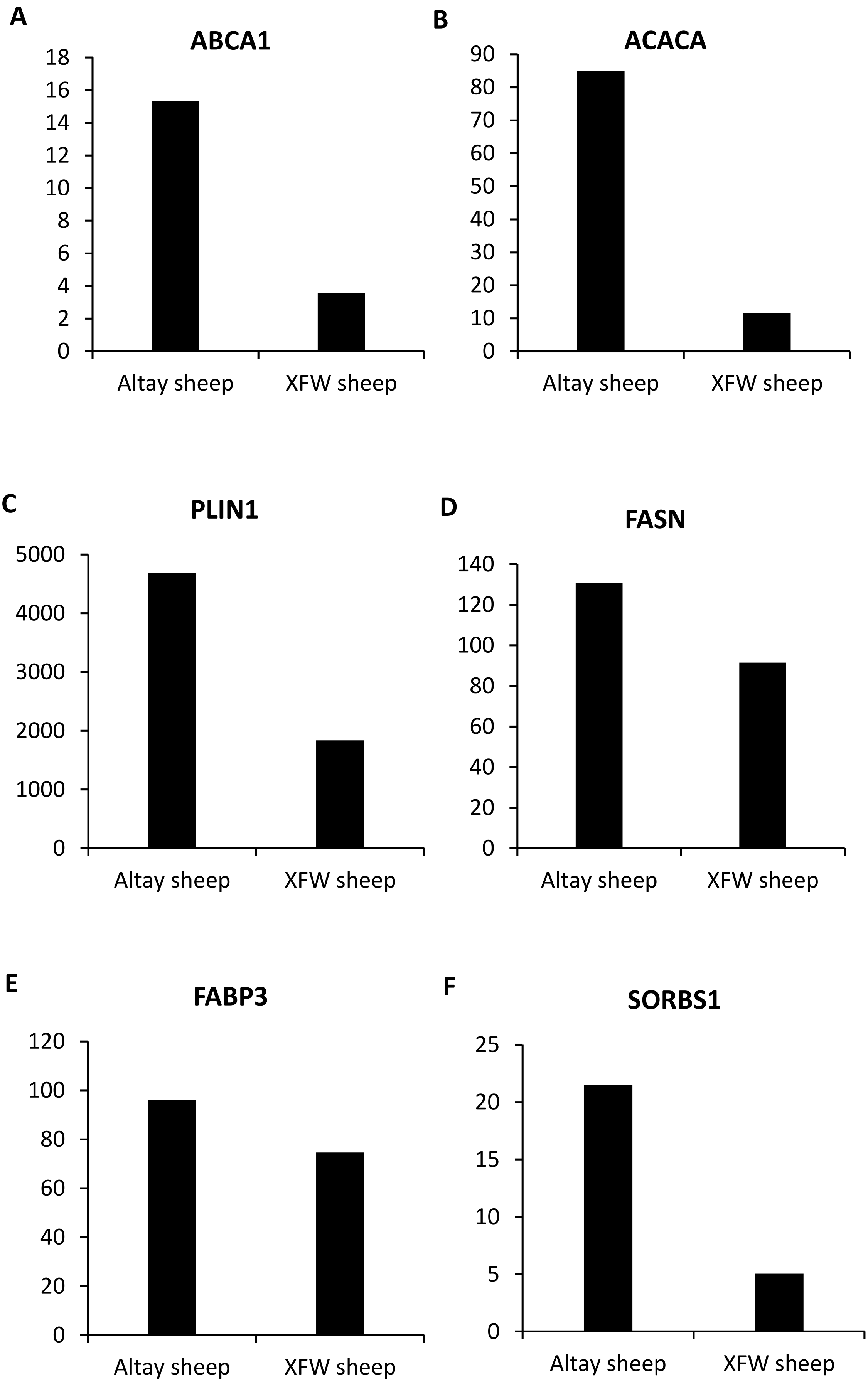
A
ABCA1
18
16
14
12
10
8
6
4
2
0
Altay sheep
XFW sheep
B
ACACA
90
80
70
60
50
40
30
20
10
0
Altay sheep
XFW sheep
C
PLIN1
5000
4000
3000
2000
1000
0
Altay sheep
XFW sheep
D
FASN
140
120
100
80
60
40
20
0
Altay sheep
XFW sheep
E
FABP3
120
100
80
60
40
20
0
Altay sheep
XFW sheep
F
SORBS1
25
20
15
10
5
0
Altay sheep
XFW sheep

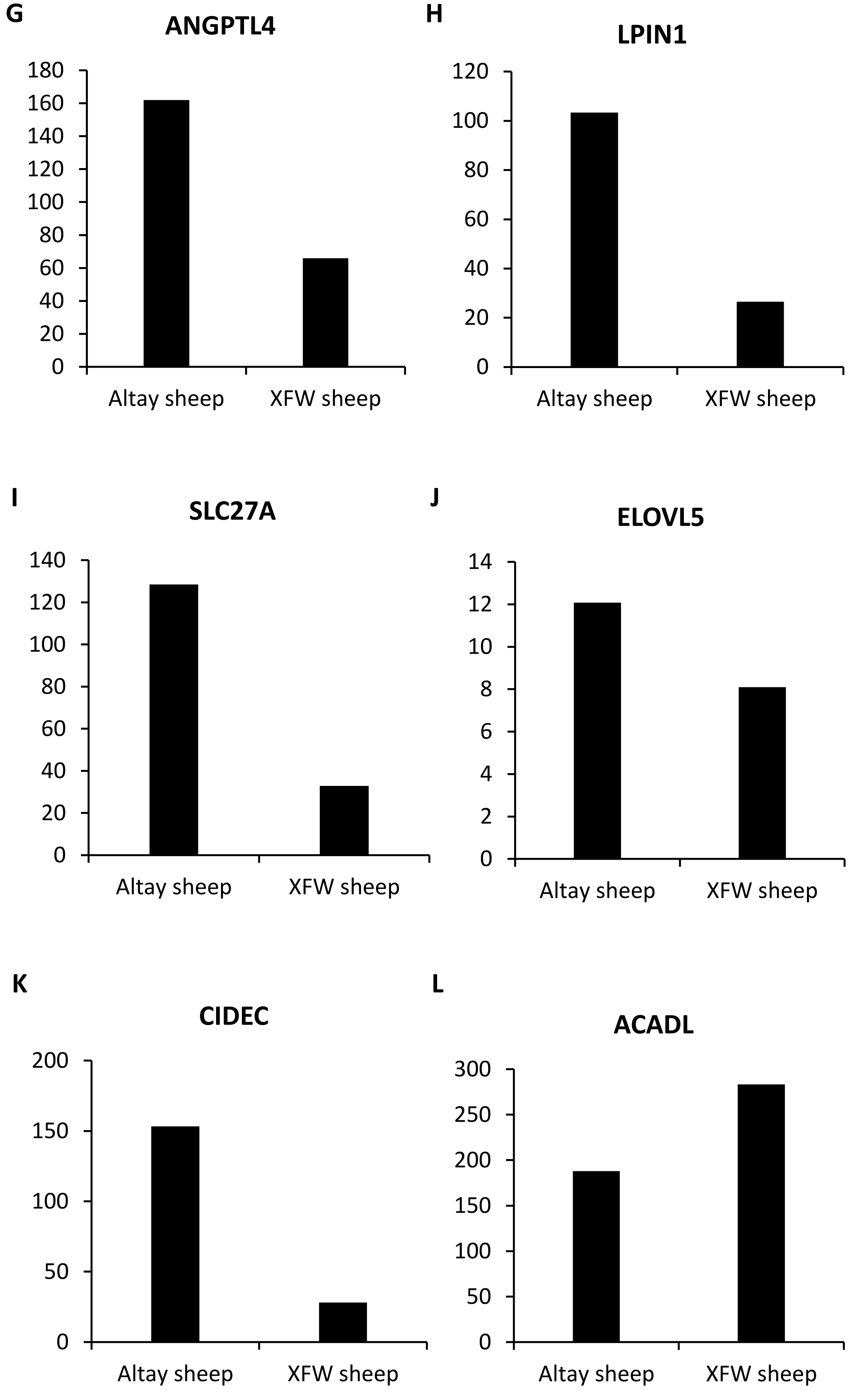
G
ANGPTL4
180
160
140
120
100
80
60
40
20
0
Altay sheep
XFW sheep
H
LPIN1
120
100
80
60
40
20
0
Altay sheep
XFW sheep
I
SLC27A
140
120
100
80
60
40
20
0
Altay sheep
XFW sheep
J
ELOVL5
14
12
10
8
6
4
2
0
Altay sheep
XFW sheep
K
CIDEC
200
150
100
50
0
Altay sheep
XFW sheep
L
ACADL
300
250
200
150
100
50
0
Altay sheep
XFW sheep

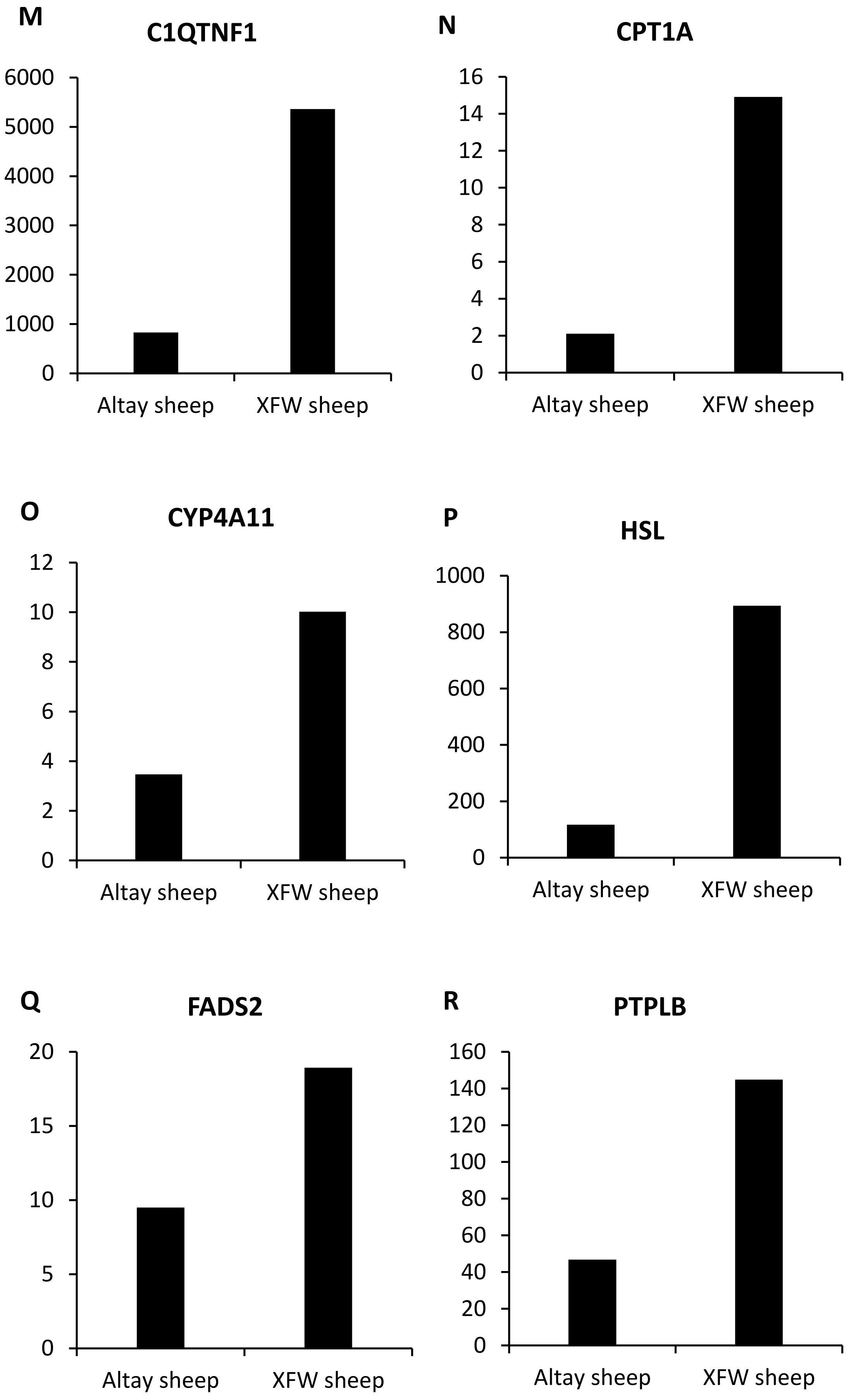
M
C1QTNF1
6000
5000
4000
3000
2000
1000
0
Altay sheep
XFW sheep
N
CPT1A
16
14
12
10
8
6
4
2
0
Altay sheep
XFW sheep
O
CYP4A11
12
10
8
6
4
2
0
Altay sheep
XFW sheep
P
HSL
1000
800
600
400
200
0
Altay sheep
XFW sheep
Q
FADS2
20
15
10
5
0
Altay sheep
XFW sheep
R
PTPLB
160
140
120
100
80
60
40
20
0
Altay sheep
XFW sheep

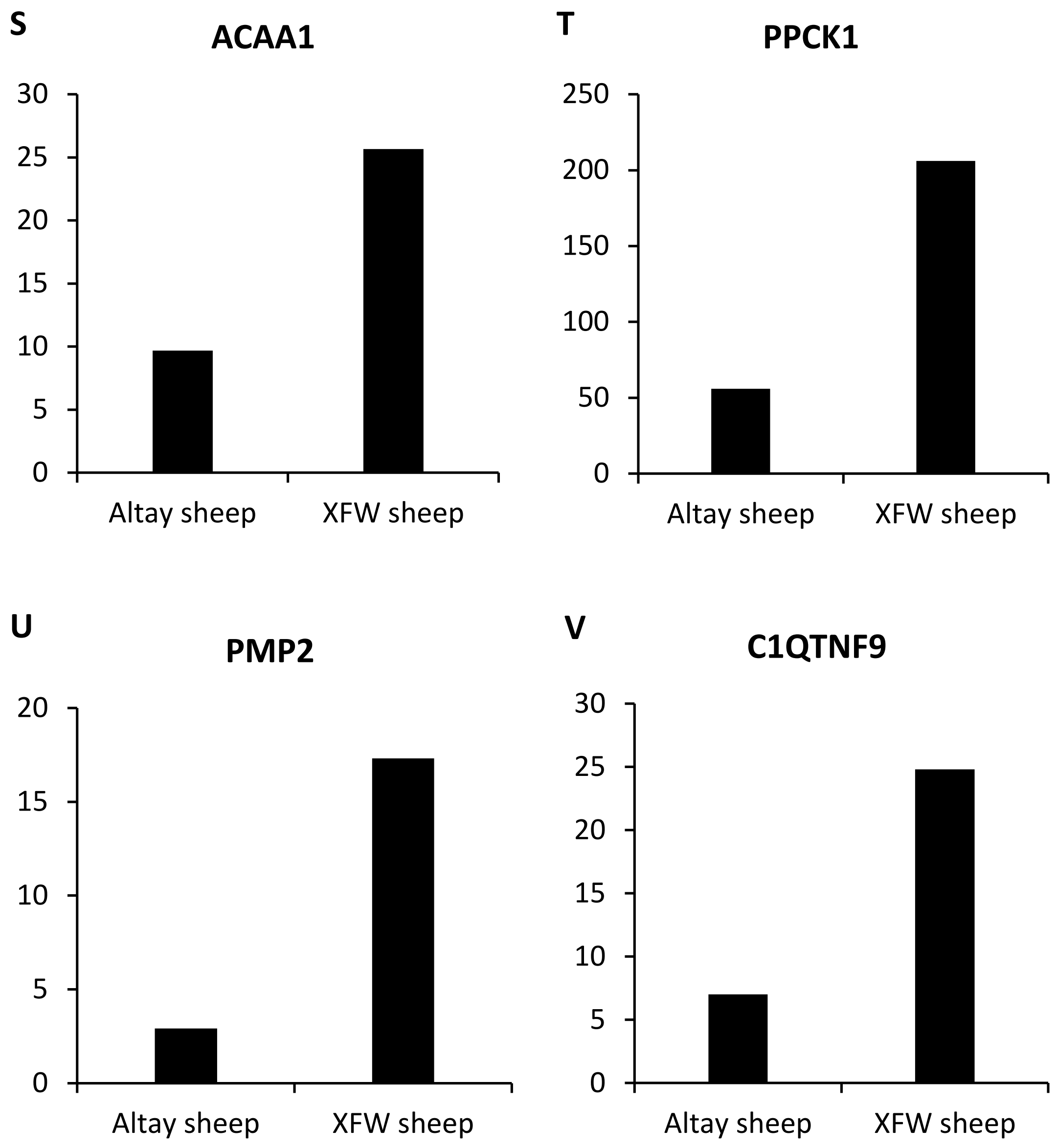


**Figure S2** Expression levels of 11 upregulated genes (a-k) and 11 downregulated genes (l-v) in tail fat tissue of Altay sheep detected by RNA-seq.
